# Investigating the Interplay between Sister Chromatid Cohesion and Homolog Pairing in Drosophila Nuclei

**DOI:** 10.1371/journal.pgen.1006169

**Published:** 2016-08-19

**Authors:** T. Niroshini Senaratne, Eric F. Joyce, Son C. Nguyen, C.-ting Wu

**Affiliations:** Department of Genetics, Harvard Medical School, Boston, Massachusetts, United States of America; Geisel School of Medicine at Dartmouth, UNITED STATES

## Abstract

Following DNA replication, sister chromatids must stay connected for the remainder of the cell cycle in order to ensure accurate segregation in the subsequent cell division. This important function involves an evolutionarily conserved protein complex known as cohesin; any loss of cohesin causes premature sister chromatid separation in mitosis. Here, we examined the role of cohesin in sister chromatid cohesion prior to mitosis, using fluorescence *in situ* hybridization (FISH) to assay the alignment of sister chromatids in interphase Drosophila cells. Surprisingly, we found that sister chromatid cohesion can be maintained in G2 with little to no cohesin. This capacity to maintain cohesion is widespread in Drosophila, unlike in other systems where a reduced dependence on cohesin for sister chromatid segregation has been observed only at specific chromosomal regions, such as the rDNA locus in budding yeast. Additionally, we show that condensin II antagonizes the alignment of sister chromatids in interphase, supporting a model wherein cohesin and condensin II oppose each other’s functions in the alignment of sister chromatids. Finally, because the maternal and paternal homologs are paired in the somatic cells of Drosophila, and because condensin II has been shown to antagonize this pairing, we consider the possibility that condensin II-regulated mechanisms for aligning homologous chromosomes may also contribute to sister chromatid cohesion.

## Author Summary

As cells grow, they replicate their DNA to give rise to two copies of each chromosome, known as sister chromatids, which separate from each other once the cell divides. To ensure that sister chromatids end up in different daughter cells, they are kept together from DNA replication until mitosis via a connection known as cohesion. A protein complex known as cohesin is essential for this process. Our work in Drosophila cells suggests that factors other than cohesin also contribute to sister chromatid cohesion in interphase. Additionally, we observed that the alignment of sister chromatids is regulated by condensin II, a protein complex involved in the compaction of chromosomes prior to division as well as the regulation of inter-chromosomal associations. These findings highlight that, in addition to their important individual functions, cohesin and condensin II proteins may interact to organize chromosomes over the course of the cell cycle. Finally, building on prior observations that condensin II is involved in the regulation of somatic homolog pairing in Drosophila, our work suggests that the mechanisms underlying homolog pairing may also contribute to sister chromatid cohesion.

## Introduction

It is well recognized that the three-dimensional organization of interphase nuclei is non-random and can affect gene expression, development, and numerous other processes [1–4]. In addition to cell-type specific interactions between and within chromosomes [5,6], nuclear organization is shaped by chromosome-wide changes in structure that are inherent to the process of nuclear division. For instance, in addition to condensing their chromosomes into the compact forms found in metaphase, mitotically dividing cells double their DNA content and thus their chromosome number during S-phase. Diploid cells therefore transition from a G1 phase with two copies of each chromosome, called the maternal and paternal homologs, to a G2 phase with four copies of each chromosome, each homolog having been replicated to form a set of sister chromatids. Importantly, sister chromatids are held together by physical connections, beginning in S-phase and continuing through G2 into mitosis, that are critical for ensuring that the two chromatids ultimately segregate into different daughter cells [7–9]. Remarkably, these connections, known as cohesion, exist in G2 amidst a variety of other inter- and intra-chromosomal interactions and yet are uniquely maintained between sisters. This study focuses on mechanisms contributing to cohesion, defined as the connection between sister chromatids from the time of DNA replication until cell division [7,8]. In particular, we explore the possibility that cohesion may also reflect contributions from mechanisms in somatic cells that pair maternal and paternal chromosomes, which, like sister chromatids, share sequence homology (reviewed by [10]).

Sister chromatid cohesion is known to require a highly conserved, essential group of proteins known as the cohesin complex [11–13]. This complex consists of two members of the structural maintenance of chromosomes (SMC) protein family, Smc1 and Smc3, a kleisin protein called Rad21/Scc1, and an associated protein known as Stromalin/Scc3 (reviewed by [7,14]). The association of cohesin with chromatin is regulated in a cell-cycle-dependent manner, starting with the loading of cohesin during the G1/S transition in yeast [12,15] and even earlier in vertebrates [13,16,17]. The establishment of cohesion during S-phase is essential for proper cohesin function in mitosis [13,18–20]. Once chromosomes have aligned at the metaphase plate in mitosis, Rad21 is cleaved and cohesin dissociates from the chromatin, allowing sister chromatid separation [21–25]. Consistent with this, loss of cohesin leads to premature sister chromatid separation in mitosis [11–13,26–29]. Structurally, the cohesin complex forms a ring-shape [30–32], and artificially sealing the ring by chemical cross-linking prevents cohesin dissociation from DNA [33,34]. Based on these and other data, several models for how cohesin holds sister chromatids together have been proposed [30,32–40]. For example, a single cohesin molecule could encircle two sister chromatids, or cohesin molecules could bind individual chromatids and then self-associate. Importantly, as cohesin proteins have also been shown to participate in gene regulation, chromatin looping, and DNA repair (reviewed by [41]), the mechanism of cohesin activity may differ across its different functions [14,42,43] and depend to varying degrees on contributions from other proteins or other types of inter-chromosomal interactions.

The requirement of cohesin proteins for maintaining proper cohesion of metaphase chromosomes is well established in multiple organisms [11–13,26–29]. Interestingly, there is evidence suggesting that additional mechanisms contribute to cohesion as well (reviewed by [44], see also [45,46]). For example, sister chromatid separation at the rDNA locus during mitosis in *S*. *cerevisiae* requires the activity of other proteins in addition to cohesin cleavage, suggesting that this locus has connections between sisters that are independent of cohesin [47–51]. This cohesin-independent cohesion is region-specific, however, as other loci show sister chromatid separation soon after or even before the completion of DNA replication in the absence of cohesins [12,44,52]. Similarly, the extent of premature sister chromatid separation observed in metaphase in the absence of cohesin varies across chromosomes in Xenopus [29,53], chicken [27], and human cells [54]. These observations raise questions regarding the nature of cohesin-independent connections between sister chromatids, why the cell might have such connections in addition to those mediated by cohesin proteins, and how cohesin-independent mechanisms might contribute to other inter-chromosomal associations and nuclear organization throughout the cell cycle.

The nuclei of *Drosophila melanogaster* and other Dipteran insects present a unique situation, where sister chromatid cohesion is not the only association between chromosomes that are homologous to each other. In these insects, maternal and paternal homologs show robust pairing in somatic cells throughout development (reviewed by [55]), occurring in different stages of the cell cycle and in many different tissues. Additionally, this pairing impacts gene expression through mechanisms known as transvection (reviewed by [56–58], see also [59–63]). Therefore, homolog pairing is a prominent and functional feature of Dipteran nuclear organization. Since cohesion and homolog pairing are both interactions between chromosomes sharing sequence homology, we have asked whether the mechanisms underlying homolog pairing may also contribute to the cohesion of sister chromatids.

**Fig 1:**
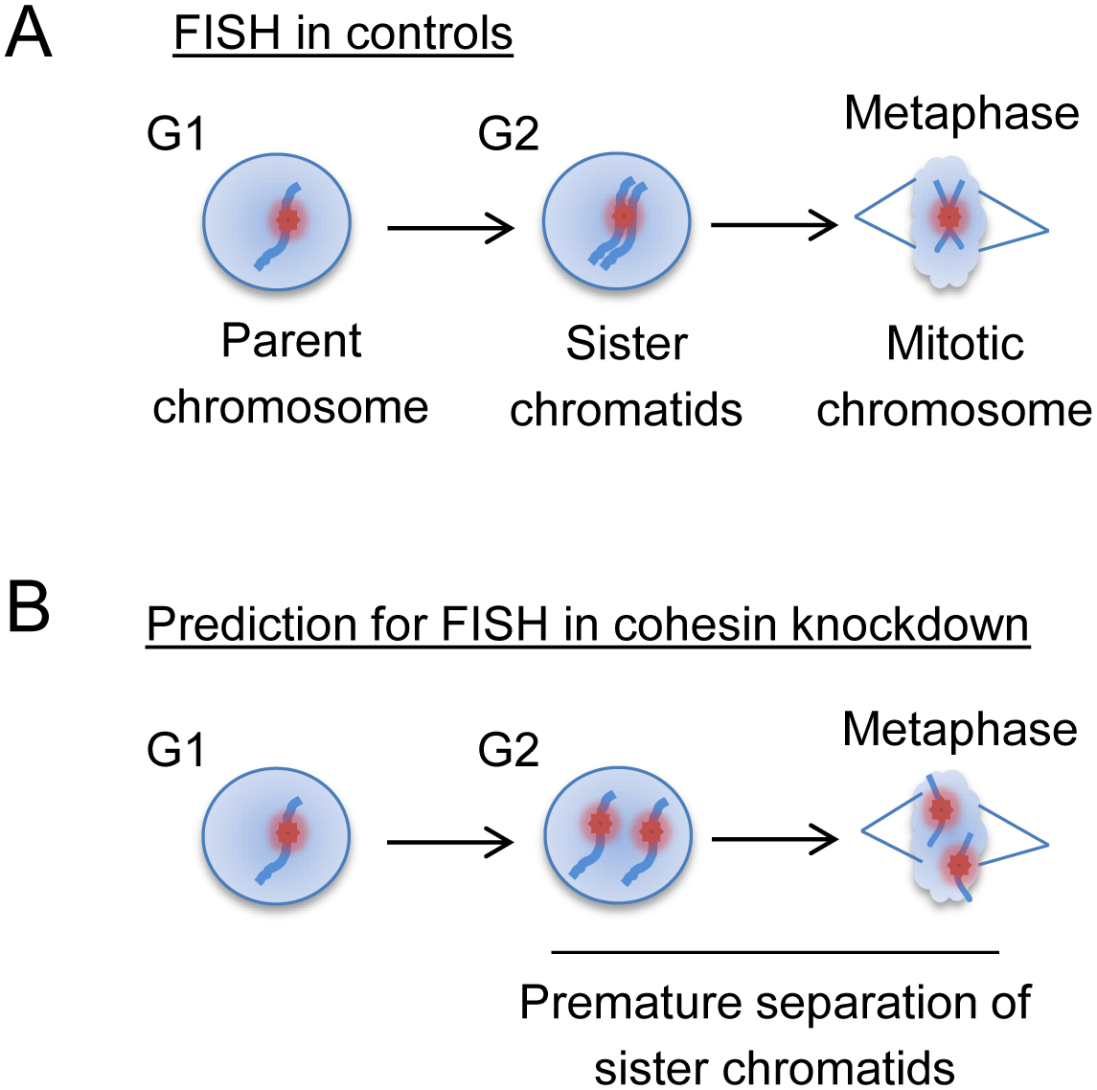
Cohesin knockdown was expected to cause sister chromatid separation in G2 and in metaphase. (A) When a specific DNA sequence is targeted by fluorescence *in situ* hybridization (FISH) in Drosophila cells, most cells show a single FISH signal (red spot) in interphase because of homolog pairing and sister chromatid cohesion. (B) Knockdown of cohesin was expected to cause sister chromatid separation and thus an increase in the number of FISH signals both in G2 and in metaphase. Note that only one homolog is shown for clarity; in reality, multiple homologs may be present, and an increase in the number of FISH signals would be expected if there was homolog unpairing, loss of sister chromatid cohesion, or both.

The initial observations leading to our studies were obtained during a genome-wide RNAi screen in cultured Drosophila cells for genes involved in somatic homolog pairing and other forms of interphase nuclear organization. This screen assayed pairing at two distinct heterochromatic loci using high-throughput fluorescence *in situ* hybridization (Hi-FISH) [64], where a single FISH signal indicated that all copies of the targeted locus were in close proximity to each other, and RNAi knockdowns leading to more or fewer FISH signals identified genes that were candidates for promoting or antagonizing pairing respectively [64]. We expected that cohesin knockdown would increase the number of signals observed in FISH assays because of sister chromatid separation (Fig 1). Such assays also tested whether cohesin contributes to homolog pairing; for example, cohesin molecules may encircle or otherwise spatially restrict homologs as well as sisters, or the presence of cohesion between sister chromatids may facilitate alignment and/or recombination of homologs, as occurs during meiosis in many organisms [65,66]. Surprisingly, cohesin proteins were not among the 105 genes identified in the screen. This was our first indication that Drosophila cells might have cohesin-independent pairing of homologous chromosomes and cohesion of sister chromatids.

In contrast, the screen did identify multiple components of another SMC protein complex, condensin II, as anti-pairers [64]. Condensin II, as well as another condensin complex, called condensin I, are involved in chromosome compaction prior to mitosis in many organisms [67,68]. In Drosophila, condensin II had previously been shown to antagonize homolog pairing and transvection *in vivo* [69]. Interestingly, we also found that the SCF ubiquitin ligase component Slmb promotes pairing and, consistent with the negative regulation of condensin II by Slmb through ubiquitination of the condensin II subunit Cap-H2 [70], the reduction of homolog pairing caused by Slmb knockdown is dependent on condensin II [64,70]. These and other studies of condensin II and its regulators highlight the importance of condensin II levels for proper nuclear organization [71–74]. Interestingly, condensin components have also been implicated in the regulation of sister chromatid cohesion in organisms where homolog pairing is not prevalent, including in budding yeast [47–51,75,76]. These findings suggest that homolog pairing in flies might be mechanistically related to cohesin-independent cohesion in other species, and that, in Drosophila, the mechanisms that act between sister chromatids may be similar to, or the same as, those that act between homologs. This model has also been proposed by Ono *et al*. based on studies demonstrating that condensin II in human cells begins resolving sister chromatids in S-phase [77]. Here, we present data showing that the cohesion of sister chromatids in G2 Drosophila cells can be maintained with little to no cohesin protein, and that knockdown of pairing regulators such as Slmb and Cap-H2 reveal a phenotype following cohesin loss. These results are consistent with what one might expect if the mechanisms of homolog pairing were also contributing to sister chromatid cohesion.

## Results

### Homolog pairing and sister chromatid cohesion at heterochromatic loci in Drosophila interphase cells can be maintained with little to no cohesin

In our previously published screen for genes involved in homolog pairing [64], we applied Hi-FISH to tetraploid Kc_167_ cells in 384-well plates seeded with a whole-genome RNAi library and assayed pairing at two pericentric heterochromatic loci, one consisting of the 359 satellite repeats [78] on the X chromosome and the other consisting of the dodeca satellite repeats [79,80] on chromosome 3. In this study, the extent of homolog pairing was defined operationally as the percentage of nuclei in a population with one FISH signal per locus, and because a single signal would also require sister chromatid cohesion, our assay of homolog pairing also reflected sister chromatid cohesion in interphase. As no cohesin subunits or associated proteins were identified in the screen, our screen suggested that homolog pairing as well as sister chromatid cohesion can occur with little to no cohesin protein at the two loci assayed.

**Fig 2:**
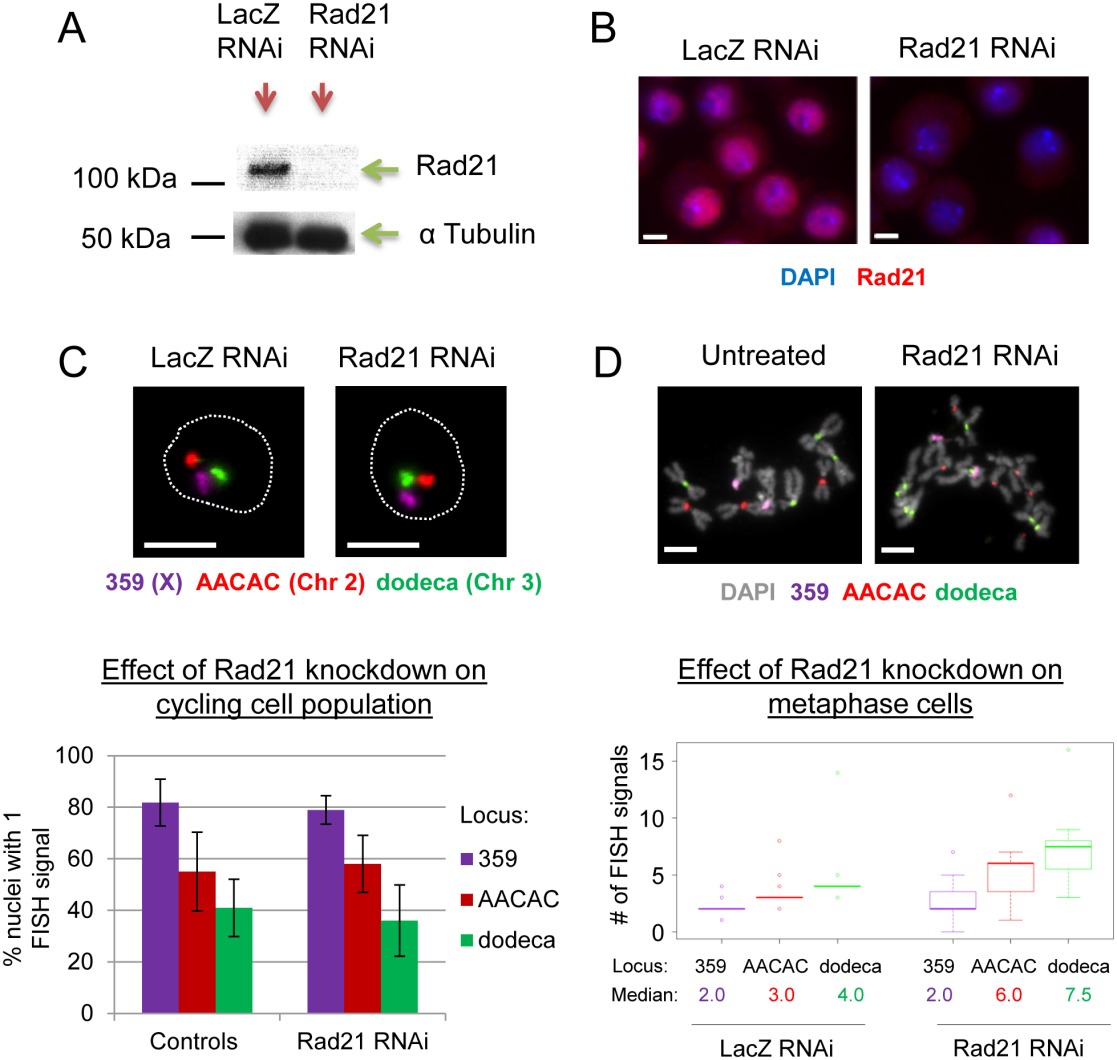
Cohesin knockdown in Drosophila cells disrupts sister chromatid cohesion in mitosis, but not interphase. (A) Western blot showing that, after four days of RNAi in S2R+ cells, Rad21 knockdown is efficient. (B) Immunofluorescence for Rad21 confirms knockdown at the level of individual cells. (C) FISH targeting pericentric heterochromatin of the X chromosome and chromosomes 2 and 3 shows that Rad21 knockdown does not increase the number of FISH signals observed in interphase (dotted line, perimeter of DAPI signal). Shown are the percentages of nuclei with single FISH signals in Rad21 RNAi-treated cells compared to controls (controls include untreated cells and LacZ RNAi). Data shown are the means of 8–10 independent trials; error bars = SD; n>190 nuclei per knockdown per trial; differences between controls and Rad21 RNAi were not significant by Fisher’s exact test (calculated for each trial) and by Student’s t-test for pooled averages from multiple trials (P = 0.3909, P = 0.6214, P = 0.4526 for 359, AACAC and dodeca, respectively). (D) Metaphase spreads with FISH show that Rad21 knockdown causes sister chromatid separation for AACAC and dodeca, increasing the number of FISH signals per locus in mitosis (for discussion of 359, see main text). Boxplot shows results from a single trial (n≥32 mitotic nuclei per knockdown; differences between controls and Rad21 RNAi treated cells were significant by Mann-Whitney U test, P<0.0001 for AACAC and dodeca, P = 0.0126 for 359). For another independent trial, see S10 Fig. All scale bars represent 5 μm.

**Fig 3:**
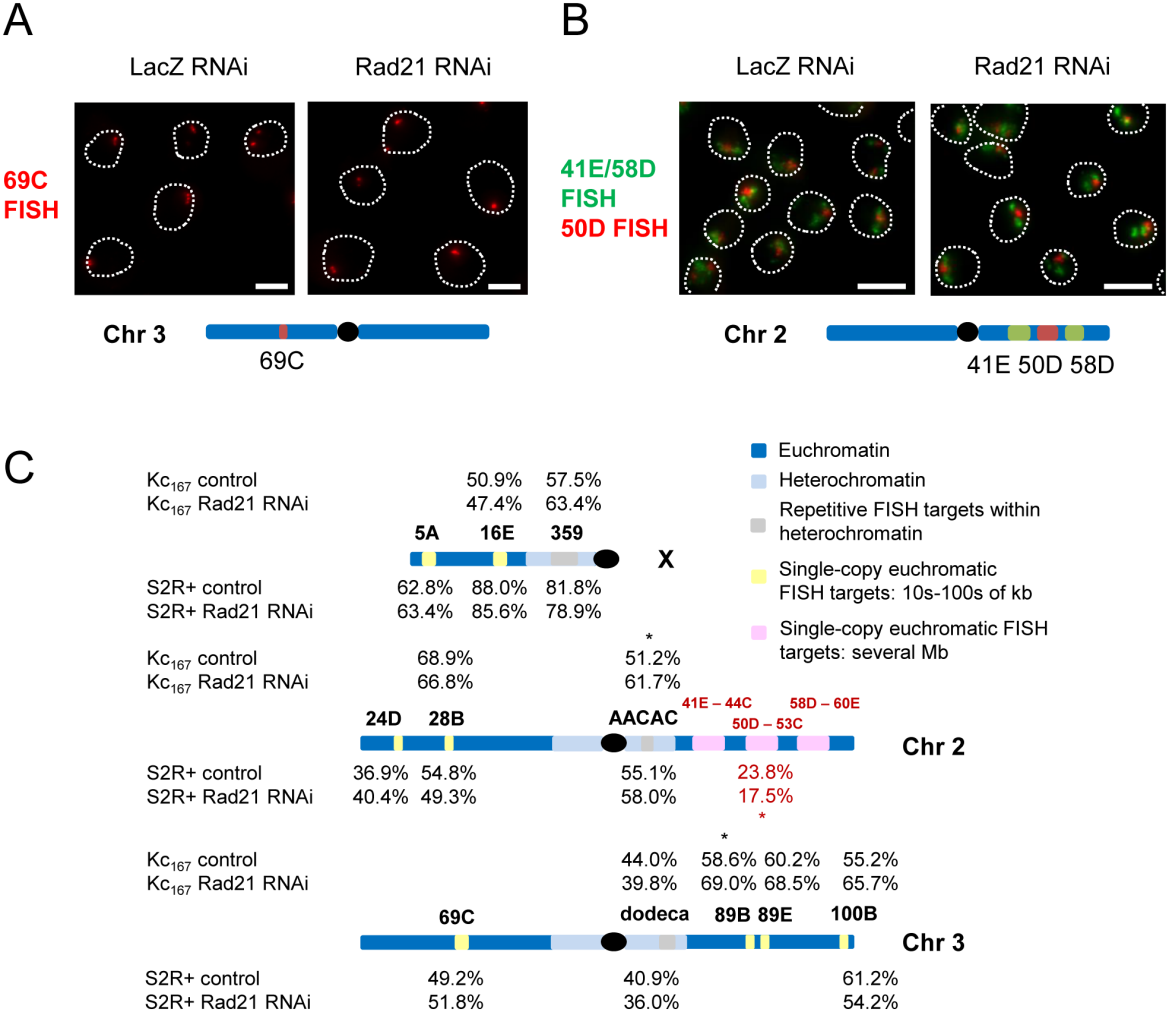
Homolog pairing and sister chromatid cohesion in interphase remain unaffected genome-wide after knockdown of Rad21. (A) FISH targeting the 69C euchromatic locus (chromosome 3L, 674 kb) shows that the number of FISH signals per nucleus did not increase following Rad21 RNAi (dotted line, DAPI perimeter; scale bar = 5 μm). (B) FISH targeting the 41E, 50D and 58D loci (chromosome 2R, total of 8.4 Mb) shows that the size of the FISH signals did not increase following Rad21 RNAi (dotted line, DAPI perimeter; scale bar = 10 μm). (C) Schematic showing the three major chromosomes of Drosophila and summarizing the effects of Rad21 knockdown, with heterochromatic FISH targets highlighted in grey, smaller euchromatic FISH targets (tens to hundreds of kilobases) highlighted in yellow, and larger euchromatic FISH targets (several megabases) highlighted in pink. The sizes of these targets are as follows: 359 (estimated size ~11 Mb [85]), AACAC (size unknown), dodeca (size unknown), 5A (672.0 kb), 16E (700.0 kb), 24D (490.6 kb), 28B (680.0 kb), 69C (674.0 kb), 89B (49.7 kb), 89E (49.7 kb), 100B (462.3 kb), 41E-44C (3.1 Mb), 50D-53C (2.7 Mb), 58D-60E (2.6 Mb). For the grey and yellow targets, percentages represent the proportion of nuclei in a cycling cell population with a single FISH signal, while, for the pink targets, percentages represent the proportion of the nuclear area occupied by the FISH signals. Results for Kc_167_ are shown above each chromosome while those for S2R+ cells are shown below. Data are from single trials except for 359, AACAC, dodeca, 16E, and 100B, where the percentages are the mean results from 2 or more trials; for all loci, n≥150 nuclei per trial. Significance was assessed using Fisher’s exact test (calculated separately for each trial), except for the pink targets, when a t-test was used to compare FISH signal areas (asterisks, P<0.05 for difference between control and Rad21 knockdowns in each trial). A simple separation of sister chromatids would be expected to cause a decrease in the percentage of nuclei with a single FISH signal, or an increase in the area of FISH signals; we observed neither (see main text for details).

Given the unexpected nature of these findings, we began our studies by determining whether our failure to identify cohesin in the screen was due to an artifact of culturing cells in 384-well plates and using Hi-FISH as part of the screening protocol and/or due to incomplete knockdown by RNAi. To do so, we performed studies using conventional slide-based FISH in both Kc_167_ cells and another Drosophila cell line, S2R+. Under these conditions, we found that, compared to levels in control cells, the mRNA levels of Rad21, Smc1, Smc3, and Stromalin (SA) were reduced by 83%, 87%, 92%, and 79%, respectively, following four days of RNAi, which was the duration of RNAi knockdown used in the screen (S1 Fig). Consistent with this, Rad21 protein levels were also depleted when assayed by Western blot and immunofluorescence in both S2R+ (Fig 2A and 2B) and Kc_167_ cells (S2 Fig); by Western, the efficiency of Rad21 knockdown at the protein level was estimated to be 88–89% (S3 Fig). Importantly, no significant reduction in the percentage of nuclei with only a single FISH signal was observed in cohesin RNAi-treated cells as compared to controls when we targeted FISH to 359 (81.8% versus 78.9%, P = 0.4526) and dodeca (40.9% versus 36.0%, P = 0.3909), as well as the AACAC pericentric heterochromatic repeat locus [81] on chromosome 2 (55.0% versus 58.0%, P = 0.6214) (Fig 2C; values are for S2R+ cells, but lack of a significant reduction was also observed with Kc_167_ cells, see Fig 3 for more details). We also tested simultaneous knockdowns of multiple cohesin proteins (S4A Fig) and longer RNAi treatments (S4B Fig) and found no consistently significant effects on the percentage of nuclei with single FISH signals. Although these data do not rule out degrees of sister chromatid separation that cannot be detected by diffraction-limited light microscopy (Materials and Methods), they nevertheless argue that sister chromatids are able to remain in relatively close proximity with little to no cohesin protein.

Efficient knockdown of cohesin was also confirmed by the observation of premature sister chromatid separation in metaphase following cohesin RNAi (Fig 2D and S5 Fig). That is, after knockdown of Rad21, Smc1, or Smc3, sister chromatids appeared as single chromosomes rather than the pairs of connected chromatids that are normally observed in metaphase spreads (S5 Fig). We then extended this analysis, focusing on Rad21 because the extent of sister chromatid separation observed in metaphase following Rad21 knockdown was more severe than that observed following knockdown of either Smc1 or Smc3 (S5 Fig). Here, we first determined the copy number of each chromosome in control cells by performing FISH on metaphase spreads, confirming that our S2R+ cell line has an irregular but stable karyotype, with two copies of the X, three copies of chromosome 2, and four copies of chromosome 3 (Fig 2D). We then applied FISH after Rad21 knockdown and observed an increase in the number of FISH signals at AACAC and dodeca (P<0.0001 for both); additionally, the median numbers of FISH signals increased from 3.0 and 4.0 signals in control cells at AACAC and dodeca, respectively, to 6.0 and 7.5 in Rad21 RNAi-treated cells (Fig 2D). While we cannot rule out contributions from aneuploidy, the approximate doubling of the median along with the observation of separated chromatids is strongly indicative of mitotic sister chromatid separation after cohesin knockdown. These findings further suggest that at this stage of the cell cycle, there is little cohesin-independent cohesion, as has been previously observed [82]. Importantly, this loss of cohesion in mitotic cells was unlikely to have had a significant impact on the overall percentage of nuclei with single FISH signals in our screen or in subsequent experiments, since mitotic nuclei represent only a small percentage of a cycling population; we found the mitotic index following Rad21 knockdown to be around 7%, consistent with published results [28]. The fact that we do not see massive levels of aneuploidy following cohesin knockdown suggests that there may be residual cohesin contributing to segregation to some degree, despite being insufficient to keep sister chromatids tethered together during metaphase. Alternatively, it is possible that cohesin-independent mechanisms contribute to segregation. There is evidence to suggest that segregation is not completely disrupted following cohesin cleavage; for example, studies using TEV-protease to induce cleavage of cohesin in Drosophila have found that prematurely separated sister chromatids often segregate to opposite poles [83], and that single chromatids form stable attachments to the spindle despite lacking tension provided by cohesin between sister chromatids [84].

Note that, while cohesin knockdown approximately doubled the median numbers of FISH signals in mitotic cells at AACAC and dodeca, it was not sufficient to completely disrupt cohesion at 359; while there was sometimes a significant increase in the number of FISH signals at 359 (P = 0.0126), indicative of some sister chromatid separation, the median was unchanged, remaining at 2.0 and indicating that sister chromatids remained connected in many cells (Fig 2D; note that significance was not always achieved for 359, see S10 Fig). It is possible that this result could be explained by residual cohesin protein that is present at the 359 locus. Alternatively, 359 may retain a cohesin-independent connection between sister chromatids even in mitosis. Either interpretation suggests that the 359 locus requires less cohesin than do other loci to maintain cohesion in mitosis. Intriguingly, the 359 locus is proximal to the rDNA locus on the Drosophila X chromosome [85], raising the possibility that its cohesion is influenced by that of the rDNA; as mentioned earlier, the rDNA locus of *S*. *cerevisiae* displays cohesin-independent cohesion [47–51].

The sister chromatid separation observed in mitosis but not in interphase following Rad21 knockdown raises the possibility that the former reflects, at least to some extent, physical forces that disjoin sister chromatids in mitosis. As such, sister chromatid cohesion might be maintained in G2 not because sisters can be held together in the absence of cohesin, but because they are not being actively pulled apart. To address this possibility, we prepared metaphase spreads in both the absence and presence of colchicine, an inhibitor of microtubule polymerization. Addition of colchicine increased the number of metaphase spreads that were obtained from 1.25% to 3.88% of cells without RNAi, and from 1.86% to 4.84% of Rad21 RNAi-treated cells (S1 Table), consistent with the role of colchicine in inhibiting mitosis by blocking spindle assembly. Importantly, we found that colchicine does not significantly alter cohesion in metaphase following Rad21 knockdown (S1 Table); the percentage of metaphase spreads with intact sister chromatid cohesion after knockdown was 25% without colchicine and 34% with colchicine (P = 0.1570). These values are both significantly less than the 77% of cells without RNAi having intact cohesion in the presence of colchicine (P<0.0001). These results argue that loss of cohesion in mitosis cannot be explained by spindle assembly, alone, and thus suggests that the failure of Rad21 knockdown to separate sister chromatids in G2 may entail another aspect of interphase cells, such as homolog pairing.

To better understand the progression of cohesin depletion, we performed a timecourse of Rad21 knockdown in Kc_167_ cells and observed premature sister chromatid separation in mitotic cells as early as the third day following RNAi treatment (S6C Fig). As Kc_167_ cells complete the cell cycle in 24–30 hours [86], this observation argues that populations of cells that have been treated with RNAi for four or more days should have experienced cohesin depletion for the duration of at least one cell cycle. These studies also enabled us to address whether our inability to observe an effect of cohesin knockdown in interphase cells resulted from inadvertent disruption of the cell cycle; for example, arrest in G1, prior to S phase, would necessarily preclude sister chromatid separation. Evidence against this explanation was the fact that, while Rad21 knockdown caused an increased mitotic index, cells continued cycling, albeit with a delay as compared to control cells (S6A Fig), consistent with published results [28,84]. In addition, both FACS analysis (S6B Fig) and immunofluorescence for cyclin B, a protein that is expressed from S-phase through G2/M-phase [87–90] (S12B Fig), confirmed that at least two-thirds of the cell population is in G2 following cohesin knockdown. These observations argue that the apparent maintenance of cohesion following cohesin knockdown cannot be explained by a paucity of G2 nuclei. Thus, in conjunction with the findings described above, our studies indicate that the 384-well FISH format cannot explain why cohesin was not identified as a candidate gene in our screen [64], and further, that neither inefficient knockdown nor a paucity of G2 nuclei can explain why cohesin RNAi treatment does not disrupt sister chromatid cohesion or homolog pairing in interphase cells. As such, our studies suggest that either the low levels of residual cohesin protein remaining following RNAi treatment are sufficient for cohesion in G2 cells and/or that additional cohesin-independent mechanisms contribute to cohesion in interphase.

### Interphase cohesion and homolog pairing can be maintained with little to no cohesin genome-wide

As the three loci we initially examined by FISH were all located within pericentric heterochromatin, it was possible that the reduced requirement for cohesin we observed in interphase cells was specific to repetitive or heterochromatic sequences. Therefore, we used FISH to target eleven euchromatic regions in a variety of genomic locations following Rad21 knockdown (Fig 3). Applying Oligopaint [91] FISH probes to control and Rad21 RNAi-treated cells, we targeted eight euchromatic loci ranging in size from tens to hundreds of kilobases and representing all major Drosophila chromosomes: 5A (X chromosome, target size 672.0 kb), 16E (X, 700.0 kb), 24D (2L, 490.6 kb), 28B (2L, 680.0 kb), 69C (3L, 674.0kb), 89B (3R, 49.7 kb), 89E (3R, 49.7 kb) and 100B (3R, 462.3 kb) (Fig 3A and 3C). Strikingly, we did not find the percentage of nuclei with a single FISH signal to be consistently and significantly reduced at any locus following Rad21 knockdown in either Kc_167_ or S2R+ cells (Fig 3C). These data suggest that the reduced requirement of cohesin protein to maintain interphase cohesion and homolog pairing is a property of both single-copy euchromatic as well as pericentric repetitive regions.

Considering the possibility that cohesin might be required to maintain sister chromatid cohesion and homolog pairing on a more global scale in ways not obvious from the analysis of short chromosomal regions, we also tested cohesin knockdowns with Oligopaints targeting three large regions on the right arm of chromosome 2 (3.1 Mb, 2.7 Mb and 2.6 Mb) (Fig 3B). Examining a large region minimized the chances of visualizing only late-replicating regions where, in early G2, sister chromatids may not yet have formed. Additionally, large FISH targets allowed a greater dynamic range in the size of the FISH signals, permitting us to more easily measure the area of the FISH signals in maximum-Z projections, in addition to counting the number of signals. We reasoned that an assay of signal size might be more sensitive to local separation of sister chromatids and/or homologs occurring anywhere along the chromosome arm even if complete separation had not occurred. Following knockdown of Rad21, neither the number of FISH signals nor the area of the image covered by these signals showed a significant increase, contrary to what might have been expected if sister chromatids had simply separated (Fig 3C); the combined area of the FISH signals was 23.8% and 17.5% of the nuclear area in control and Rad21 RNAi-treated cells, respectively (P<0.0001 indicating a significant decrease). The decrease in signal areas we observed following Rad21 knockdown was unexpected, and could indicate an interesting role for cohesin in antagonizing compaction of chromatin, though further experiments are necessary to confirm this trend. Along these lines, it is interesting to note the significant increase in the percentage of nuclei with a single FISH signal at some loci examined with smaller FISH probe sets, specifically 89B in S2R+ cells and AACAC in Kc_167_ cells (see Fig 3C). Regardless, our data suggest that sister chromatids can maintain cohesion and homologs can remain paired across all chromosome arms with very little or no Rad21.

### Interphase cohesion following cohesin knockdown does not depend on the presence of a homologous pairing partner

**Fig 4:**
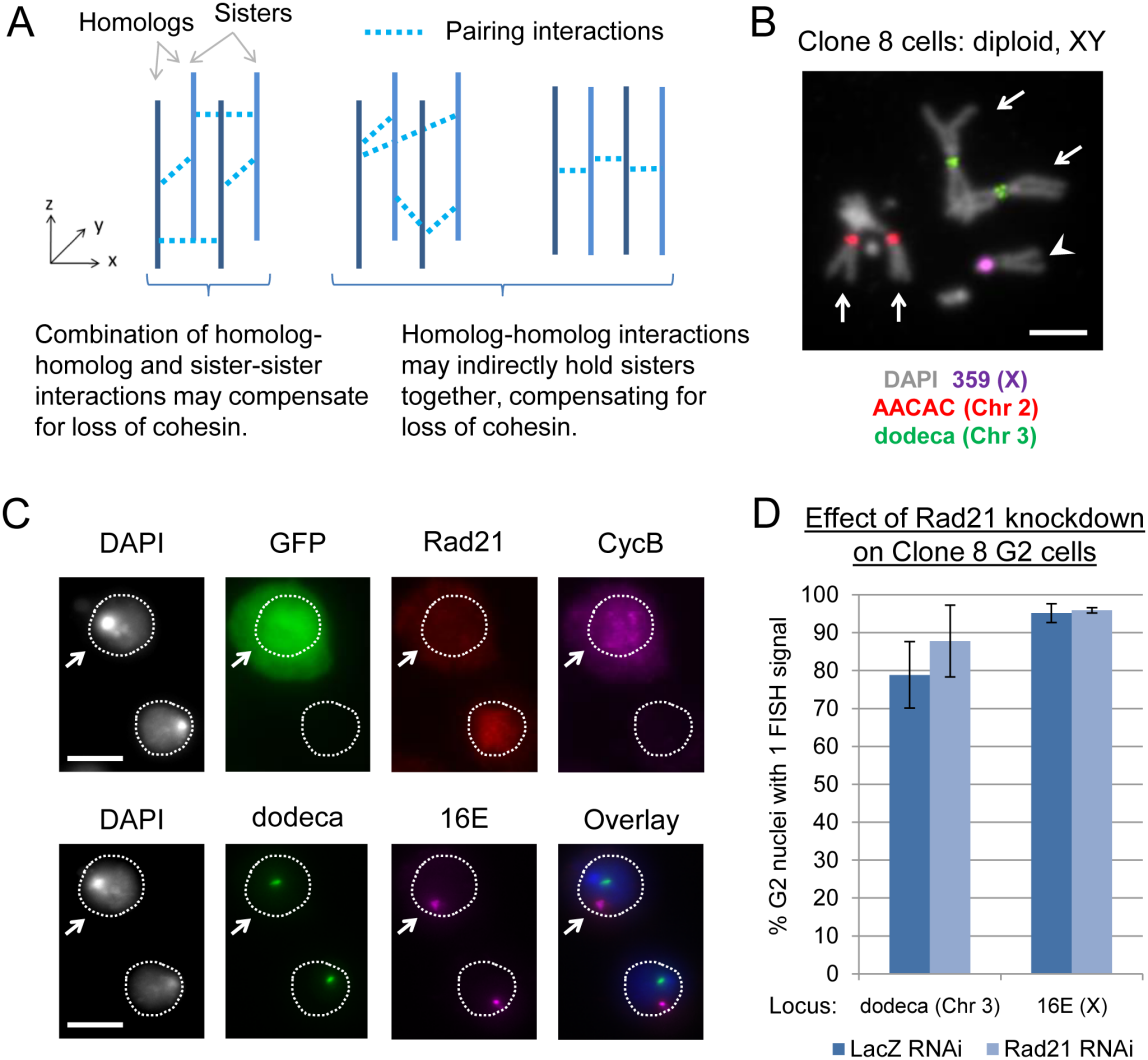
Interphase cohesion between sister chromatids following cohesin knockdown does not require the presence of a homolog. (A) Cartoon showing theoretical interactions between chromosomes. Homologs and sister chromatids can be held together by a combination of homolog-homolog and sister-sister interactions (left) or just homolog-homolog interactions that indirectly hold sister chromatids together (middle, right). (B) Karyotype of Clone 8 cells, which have two copies of the autosomes (long arrows) and a single X chromosome (arrowhead). Scale bar = 5 μm. (C) Clone 8 cells following Rad21 knockdown (top row, immunofluorescence; bottom row, FISH). GFP serves as a marker for cells transfected with dsRNA, while Cyclin B (CycB) indicates cells in the G2 stage of the cell cycle. The cell marked by the arrow is in G2 and depleted for cohesin, but FISH indicates that sister chromatid cohesion is unperturbed, both for an autosome (dodeca, 3rd Chr.) and for the X chromosome (16E). Scale bar = 5 μm. (D) Quantification of results for experiment illustrated in (C) showing percentages of nuclei with a single FISH signal at dodeca and 16E. Means represent three independent trials; error bars = SD; n≥40 per trial per knockdown. Differences between control and Rad21 knockdown cells were not significant by Fisher’s exact test (P = 0.0654 and P = 0.7992 for dodeca and 16E, respectively).

We next addressed whether homolog pairing can contribute to the cohesion of sister chromatids in interphase, reducing the requirement for cohesin proteins. For example, the mechanisms that pair homologs might also act directly between sister chromatids, holding them together even in the absence of cohesin. Alternatively, it is possible that, because homologs are paired in G1, the replication products of these chromosomes can remain closely associated in G2 without mechanisms acting directly to hold sisters together (Fig 4A). To test this second possibility, we studied a chromosome that does not have a homolog, that is, the single X chromosome in a diploid XY male cell. We reasoned that if interphase cohesion between sisters following cohesin knockdown is dependent on the presence of a homolog and/or pairing between the homologs, the X chromosome in a male cell line should display disrupted cohesion while the autosomes, which are present in two copies, should not.

For these studies we selected Drosophila Clone 8 (Cl.8+) cells, which we confirmed by karyotyping to be stably diploid and XY (Fig 4B). Given the low efficiency of RNAi in Clone 8 cells (S7 Fig), as versus Kc_167_ or S2R+ cells, we used GFP as a co-transfection marker for dsRNA. We carried out immunofluorescence for GFP, cyclin B (a G2 marker), and Rad21 to identify the cells of interest (positive for GFP and cyclin B and negative for Rad21) followed by FISH targeting region 16E on the X chromosome and dodeca on chromosome 3 (Fig 4C; Materials and Methods). Consistent with our results in other cell lines, the percentage of nuclei with a single FISH signal at dodeca was not significantly different between control G2 cells and those treated with Rad21 RNAi (78.9% and 87.8%, respectively, P = 0.0654) (Fig 4D). Remarkably, we found that sister chromatid cohesion at 16E on the X chromosome was also unaffected by cohesin knockdown, with 95.2% and 95.9% of control and Rad21 RNAi-treated G2 cells, respectively, having a single FISH signal (P = 0.7992) (Fig 4D). These observations argue that, barring intrinsic features that may be specific to the X chromosome, cohesion between sister chromatids can be maintained with little to no cohesin protein even when these chromosomes have never experienced homolog pairing. Thus, our data suggest that, whatever mechanism might be compensating for the loss of cohesin, it is not dependent on homolog pairing in the preceding G1, and therefore may initiate in S-phase/G2 and act directly between sister chromatids.

Note that 16E is located within the euchromatic arm of the X and >5 Mb away from the rDNA locus, which is positioned near the centromere. Thus, while the rDNA of the X and Y chromosomes can support local pairing [92], we consider it unlikely that pairing of the rDNA loci accounts for cohesion with little to no cohesin at 16E. That being said, it remains possible that inter-chromosomal associations occurring near the centromere might influence the organization of a chromosome arm. For example, pairing of X and Y near the centromere might lead to nonhomologous “pairing” between their arms, which could influence sister chromatid cohesion.

### Slmb, a gene regulating the pairing of homologs, also regulates the cohesion of sister chromatids in interphase

Although cohesion of sister chromatids in cohesin-depleted G2 cells may not require the presence of a homolog, it could still depend on mechanisms that also participate in homolog pairing. To test this idea, we combined cohesin knockdown with knockdown of Slmb, a gene which is required for homolog pairing; Slmb is a negative regulator of condensin II, and Slmb knockdown leads to an increased number of FISH signals and thus a decrease in the percentage of nuclei with a single FISH signal [64,70]. If Slmb is also required for cohesin-independent cohesion, we might expect simultaneous knockdown of both Slmb and cohesin to disrupt cohesion as well as homolog pairing, leading to even more FISH signals than when Slmb alone is knocked down.

**Fig 5:**
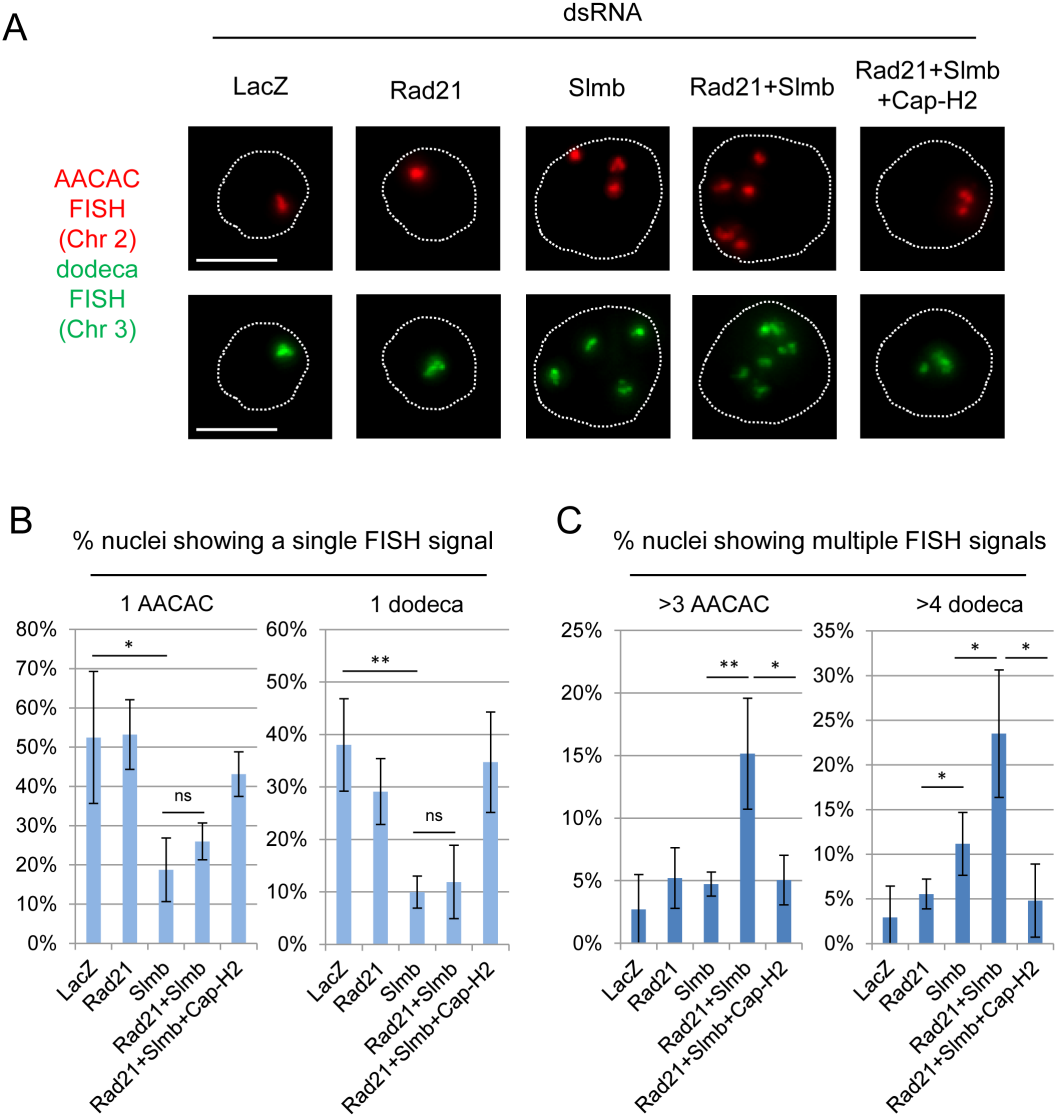
Slmb and condensin II regulate interphase sister chromatid cohesion when cohesin is depleted. (A) Nuclei from different RNAi knockdowns in S2R+ cells with FISH targeting AACAC (Chr 2) and dodeca (Chr 3). Knockdown of both Rad21 and Slmb produced more FISH signals as compared to knockdown of Slmb alone. The extra FISH signals seen in the double knockdown of Rad21 and Slmb were suppressed in a triple knockdown with Cap-H2 (dotted line, DAPI perimeter; scale bar = 5 μm). (B) Quantification of results for experiments illustrated in (A) showing percentages of nuclei with a single FISH signal, indicating nuclei where homolog pairing and sister chromatid cohesion are intact. (C) Quantification of results for experiments illustrated in (A) showing percentages of nuclei with more than 3 AACAC signals or more than 4 dodeca signals, indicating nuclei with possible sister chromatid separation in addition to homolog unpairing. In B & C, means represent 6–7 independent trials; error bars = SD; n≥100 nuclei per knockdown per trial. Significance was calculated for each trial using Fisher’s exact test and across several trials using a Student’s t-test; results from pooled tests are shown (*, P<0.05; **, P<0.0001).

We performed double knockdowns of Slmb and Rad21 in S2R+ cells and, having confirmed by qPCR that the knockdown of each gene was efficient (S8 Fig), assayed the number of FISH signals observed at pericentric heterochromatin (Fig 5A). Knockdowns of Slmb, whether alone or in combination with Rad21, reduced the percentages of nuclei with a single FISH signal at AACAC and dodeca from, respectively, 52.5% and 38.0% in control cells to 18.8% and 10.0% after Slmb knockdown and 26.0% and 11.9% following knockdown of both Slmb and Rad21 (Fig 5B). Therefore, pairing levels were similarly reduced whether we knocked down only Slmb or both Rad21 and Slmb; however, when unpairing did occur, we often observed more FISH signals when both Rad21 and Slmb were knocked down (Fig 5A and S9 Fig). In particular, the double knockdown of Rad21 and Slmb produced nuclei with four to six or more FISH signals at AACAC (Chr 2), or five to eight or more FISH signals at dodeca (Chr 3), which is noteworthy because our S2R+ cells typically carry only three copies of chromosome 2 and four copies of chromosome 3 (Fig 2D). We reasoned that the "extra" FISH signals likely represented the separation of sister chromatids and applied this approach in subsequent analyses. That is, we considered the presence of more than three AACAC FISH signals or four dodeca FISH signals in a nucleus as indicative of sister chromatid separation. As this assay requires homolog pairing as well as the separation of sister chromatids, our measure of sister chromatid separation is likely an underestimate.

Using this metric for identifying instances of sister chromatid separation, we observed that there is little sister chromatid separation following knockdown of Slmb alone; the percentages of nuclei with more than three FISH signals at AACAC or more than four FISH signals at dodeca were 4.7% and 11.2%, respectively, differing little from those of control cells (Fig 5C). In contrast, these percentages were 15.2% and 23.5% when both Rad21 and Slmb were knocked down, both values representing significant increases as compared to the outcome of knocking down Slmb alone (P<0.0001 and P = 0.0035 for AACAC and dodeca, respectively) (Fig 5C). These findings suggest that, unlike knockdown of either Slmb or Rad21 alone, the double knockdown of Slmb and Rad21 results in sister chromatid separation as well as homolog unpairing.

As the extra FISH signals could be explained by aneuploidy, we analyzed metaphase spreads following knockdowns, but did not find evidence for increased aneuploidy after double knockdown of Rad21 and Slmb as compared to knockdown of Slmb alone (S10 Fig). The extra FISH signals were also unlikely to reflect decompaction or fragmentation of heterochromatin, as double knockdowns of Rad21 and Slmb increased the number of signals at three out of five euchromatic loci studied (S11 Fig). The relatively modest effects observed at euchromatic as versus heterochromatic loci may stem from the overall higher levels of homolog pairing at euchromatin [64,93,94]. We also considered the possibility that the increase of nuclei with extra FISH signals represented the arrest of cells in mitosis, when sister chromatid cohesion is lost following cohesin knockdown. Here, we combined FISH with immunofluorescence to phosphorylated histone H3 (pH3) to identify mitotic cells [95] after knockdown of both Rad21 and Slmb, and found that exclusion of pH3-stained nuclei dropped the number of nuclei with more than three FISH signals at AACAC only slightly from 14.9% to 13.6%, which is still significantly higher than the percentage of interphase nuclei with more than three FISH signals after knockdown of Slmb alone (5.16%, P = 0.0005). Furthermore, immunofluorescence for cyclin B confirmed that the increase in the number of FISH signals in the double knockdown was not caused by an enrichment of G2 cells (S12 Fig); knockdowns decreased the proportion of G2 cells from 66.9% in control cells to 46.2% after Slmb knockdown and 41.9% after knockdown of both Rad21 and Slmb. This decrease limits the number of nuclei where sister chromatid separation is possible, and may explain why the percentage of nuclei with extra FISH signals was never more than 30%. Therefore, while we cannot rule out any contribution of aneuploidy, disorganization of heterochromatin, or cell cycle arrest, we favor the hypothesis in which the extra FISH signals in the double knockdowns of Rad21 and Slmb are caused by sister chromatid separation in interphase. This interpretation suggests that Slmb contributes to cohesion independently of cohesin. However, we also note that, even if Slmb does not regulate cohesion, any of the alternative explanations mentioned would still indicate an interesting relationship between Rad21 and Slmb and, therefore, between cohesion and homolog pairing.

### The effect of Slmb on sister chromatid cohesion is dependent on condensin II

Finally, we considered the role that Slmb plays in the negative regulation of condensin II [70] and asked whether condensin II might contribute to sister chromatid separation in our assays. Here, we asked whether the extra FISH signals we observed in the double knockdown of Rad21 and Slmb were dependent on condensin II by conducting a triple knockdown of Rad21, Slmb, and Cap-H2. Remarkably, the number of nuclei with extra FISH signals was suppressed to levels comparable to that observed for the knockdown of Rad21 or Slmb alone (Fig 5A and 5C). In particular, while the percentages of nuclei with more than three FISH signals at AACAC or more than four FISH signals at dodeca were, respectively, 15.2% and 23.5% for the double knockdown of Rad21 and Slmb, the triple knockdown of Rad21, Slmb and Cap-H2 gave significantly lower percentages, with only 5.0% and 4.8% of nuclei having extra FISH signals (P = 0.0008 and P = 0.0016 for AACAC and dodeca, respectively) (Fig 5C). Importantly, this effect was not due to a reduction in the percentage of G2 cells in the triple knockdown (S12 Fig). This suppression of the extra FISH signals by the triple knockdown also argues that the extra FISH signals observed in the double knockdown of Rad21 and Slmb were not an artifact of the double knockdown. Taken together, these results suggest that condensin II contributes to sister chromatid separation when Rad21 is compromised, raising the possibility that condensin II may contribute to sister chromatid separation under normal conditions, perhaps by removing cohesin-independent connections between sister chromatids. In light of the contributions of both condensin II and Slmb to homolog pairing [64,69–71], these data further suggest that the mechanisms mediating cohesin-independent cohesion may be similar to, or the same as, those that mediate homolog pairing.

## Discussion

Our results show that Drosophila interphase cells with little to no cohesin display levels of sister chromatid and homolog alignment comparable to that of control cells, as assayed by FISH at resolutions allowed by light-microscopy. While our studies cannot rule out that very low levels of cohesin persisted after RNAi treatment and/or that cohesin knockdown only affected a small proportion of cells, it is nevertheless surprising that the majority of cells maintain interphase chromosome organization with such low levels of cohesin (Fig 2A and 2B). If indeed the phenotypes we observed are the result of residual cohesin protein, it suggests that, at least in interphase, cohesin is not required at a high density to maintain alignment of sister chromatids along their length, while in metaphase, wild-type levels of cohesin are required for proper cohesion. This in turn raises the question of why Drosophila and other vertebrates have more cohesin loaded onto the chromosomes in G2 than in metaphase, since the majority of cohesin in these organisms is removed during prophase [96,97]. Perhaps the majority of cohesin protein present on sister chromatids in G2 participates in functions other than cohesion.

### Cohesin-independent cohesion?

As mentioned previously, the idea that there may be cohesin-independent mechanisms that contribute to segregation of sister chromatids is not new. However, in most instances where a reduced dependence on cohesin has been observed, it has been documented in mitosis and only at specific regions [44]. As for studies of interphase in organisms other than Drosophila, those that have used FISH to assay the impact of cohesin loss have detected an increase in the number of FISH signals, increased distance between signals, or abnormally shaped signals [27,98–102]. These data indicate that in most organisms, cohesin loss is sufficient to cause chromatid separation in G2, and that cohesin-independent mechanisms, if they do contribute to cohesion, do so in a locus-specific manner. Here we suggest that cohesin-independent mechanisms may be widespread in Drosophila, contributing to the pairing of homologs as well as to the cohesion of sister chromatids in G2 at 3 heterochromatic and 11 euchromatic loci, and therefore may act genome-wide. Based on our results, we cannot rule out that cohesin-independent mechanisms contributing to chromatid alignment are induced in response to cohesin knockdown. Nevertheless, these results demonstrate the potential for sister chromatids to remain aligned in interphase with little to no cohesin. It may well be no coincidence that Drosophila also supports extensive pairing of homologous chromosomes in somatic cells.

**Fig 6:**
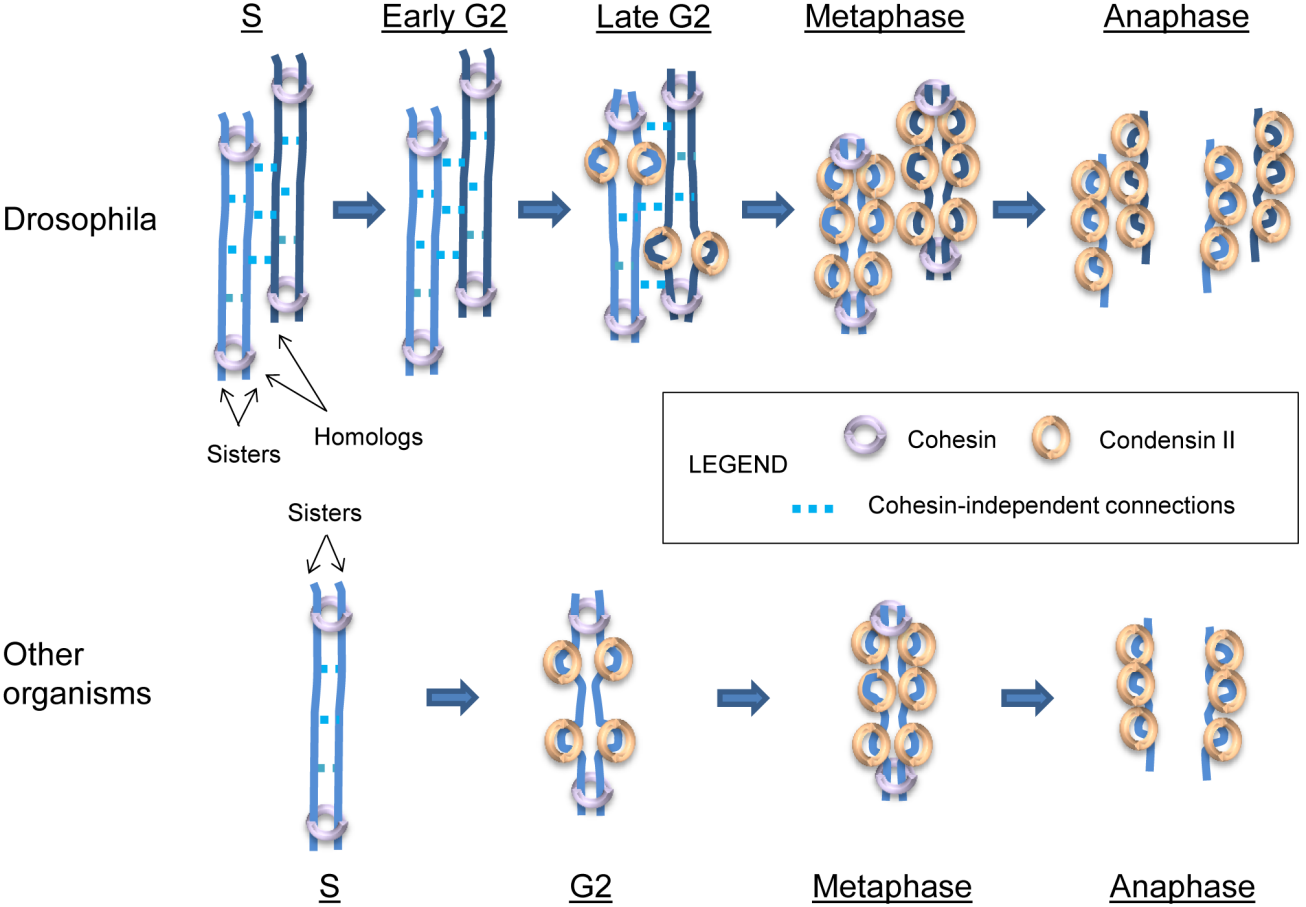
Model demonstrating how Drosophila might differ from other organisms in the extent of cohesin-independent connections between chromosomes. Hypothetical cohesin-independent connections that form between chromosomes and the timing of their removal. In this model, the cohesin-independent connections that form between sister chromatids in Drosophila (potentially, DNA catenations) also form between homologs. In other organisms, extensive cohesin-independent connections between sisters and/or homologs may not form or may be resolved more quickly (homolog not pictured). As shown, cohesin-independent connections may be resolved later in the cell cycle in Drosophila than in other organisms; alternatively, it is possible that cohesin-independent connections are formed more efficiently in Drosophila, so that they are more widespread. Condensin II may antagonize inter-homolog or inter-sister interactions in a number of ways: they may remove residual cohesin, recruit topoisomerases to remove catenations between chromosomes, or, as pictured here, compact chromosomes in *cis*, limiting their ability to participate in *trans* interactions [13,64,69,71]. Further, it is possible that condensin II cooperates with condensin I during mitosis in the process of resolving sister chromatids (see S13 Fig). Note that, in the top panel representing Drosophila, all cohesin-independent linkages are resolved by metaphase, but in theory, some connections between homologs might remain in metaphase and in anaphase. Similarly, in the lower panel representing other organisms, all cohesin-independent connections between sisters have been resolved by G2, but it is possible that they remain in certain regions.

We have also observed a genetic interaction between Rad21 and Slmb, a gene required for homolog pairing [64,70]. This finding suggests that homolog pairing and sister chromatid cohesion might be regulated by common mechanisms, consistent with a model that has been proposed by Ono *et al*. [77]. In particular, we favor a model in which the higher levels of condensin II activity caused by Slmb knockdown [70] separate sister chromatids as well as homologs in the absence of cohesin. This could happen if condensin II negatively regulates residual cohesin, or if cohesin-independent connections exist between sister chromatids as well as between homologs and condensin II antagonizes those connections (Fig 6). The latter model is supported by evidence from organisms other than Drosophila implicating condensin in the resolution of cohesin-independent connections between sister chromatids, including at the budding yeast rDNA locus [47–51]. In Drosophila, in addition to condensin II and several of its regulators being involved in homolog pairing [69–74], the condensin I subunits Barren [103–105], Cap-G [106,107], and Cap-D2 [108], as well as Smc4 (present in both complexes) [109], are required for the complete resolution of sister chromatids in mitosis. In human cells, condensin II is necessary for sister chromatid resolution beginning in late S-phase [77], and a significant amount of chromatid resolution by condensin II also takes place during prophase [110]. Consistent with these findings, our data suggest that condensin II-regulated mechanisms contribute to sister chromatid cohesion in interphase, and that this mechanism of cohesion is related to homolog pairing in Drosophila.

As for whether cohesin-independent mechanisms contribute to cohesion *in vivo*, this possibility is supported by studies showing that cohesin cleavage induced via a TEV protease in Drosophila larvae does not noticeably disrupt polytene chromosome alignment [83]. In contrast, overexpression of the condensin II component Cap-H2 disrupted polytene alignment in the same cell type [69]. It will be of great interest to determine whether similar phenotypes are observed in actively dividing fly tissues.

### Observations regarding the nature of homolog pairing

Our observations suggest that the mechanisms that act between sister chromatids may also act between homologs. This model is consistent with the idea that recognition of a pairing partner is based on DNA sequence or chromatin structure, as exemplified by the fact that chromosomal translocations can pair (e.g. [111], see also [112]), and that pairing can accommodate more than two copies of a chromosome, as has been observed most dramatically in polytene chromosomes (e.g. [113]) and polyploid cell lines (e.g. [64,93,114]). Our work also pertains to the question of whether or not cells distinguish sisters from homologs (e.g. [115]). If, as our work suggests, there are some aspects of chromosomal organization that do not distinguish sister chromatids from homologs, sister chromatids may influence gene expression beyond their contribution to chromosome copy number. For example, as homolog pairing can influence the communication between regulatory elements and promoters in *cis* as well as in *trans* [56–58], sister chromatids may be able to join and influence this dialogue [93]. Indeed, just as transvection can occur between paired homologs, so might it occur between sister chromatids, making it possible for these two forms of transvection to be synergistic or mutually inhibitory during G2 (S14 Fig). Importantly, inter-homolog communication and the contribution of sister chromatids to that process could vary by cell type, depending on the levels of cohesin-independent connections between sisters and homologs.

Our data also address the long-standing question of when in the cell cycle homolog pairing can be established. While some studies have shown that levels of homolog pairing are higher in G1 than in G2, suggesting that S-phase is a stage when pairing is more dynamic and possibly disrupted [64,116], other work shows that pairing levels are similar in G1 and G2 [93], possibly reflecting variability between cell types. Our experiments in the male diploid Clone 8 cell line suggest that cohesin-independent G2 cohesion of sisters, if it occurs, is not dependent on the presence of a homolog, which would indicate that cohesin-independent cohesion could be established *de novo* in G2. As such, perhaps the pairing of homologs can also be established at this stage. Of course, S-phase/G2 may not be the only stage of the cell cycle when homolog pairing is established; G1 homolog pairing could represent either an additional establishment event following the disruption of pairing in anaphase [117] or, theoretically, the maintenance of homologous connections from the previous cell cycle through mitosis [64,93].

### What types of connections between chromosomes may form the basis of cohesin-independent cohesion?

A major question concerns the potential nature of a cohesin-independent connection between chromosomes. There are several possibilities, including the contribution of factors, such as proteins or RNA, that function similarly to cohesin in keeping chromosomes together, but act specifically in interphase. For example, cohesion in somatic cells may involve multiple novel cohesin complexes, as is known to be the case in Drosophila meiosis [118–122]. Alternatively, cohesin-independent cohesion might involve direct connections between chromosomes themselves without any need for bridging factors, perhaps involving nontraditional base pairing or DNA catenations resulting from replication. In fact, one of the earliest models for cohesion, proposed before cohesin proteins were known, posited that sister chromatids could be held together by DNA catenations [123–125]. Consistent with this model, topoisomerase II, an enzyme that removes catenations formed during replication and other processes, is known to regulate the segregation of sister chromatids (reviewed by [126]). Furthermore, recent work has shown not only that catenations contribute to cohesion but also that cohesin may play a role in maintaining catenations [34,127]. Additionally, a complex related to cohesin and condensin known as Smc5/6 is thought to bind chromosomes in response to sister chromatid intertwinings or other forms of topological stress and facilitate their resolution in yeast [128,129]. These observations raise the questions of whether catenations might be sufficient to maintain cohesion in interphase Drosophila cells in the absence of cohesin, and how condensin II might act to resolve catenations. The role of condensin II in regulating these sorts of topological connections may be to recruit or activate topoisomerase II; alternatively, condensin II may play a role in separating chromosomes independently of topoisomerase II (reviewed by [130], see also [76,131,132]). It is also possible that the activity of condensin II in compacting chromosomes, by forming more intra-chromosomal interactions, suppresses inter-chromosomal interactions (Fig 6) [13,64,69,71]. Further experiments examining the roles of topoisomerase II and Smc 5/6 in interphase cohesion and their interactions, if any, with condensin II, will shed light on these questions.

Given the mechanistic relatedness we have observed between homolog pairing and cohesion, it is possible that homolog pairing is also mediated at least in part by DNA catenations or entanglements [59,64,93,133,134]. If so, the widespread nature of homolog pairing in Drosophila cells might imply that these cells are more permissive for the formation of catenations. For example, homologs may become catenated when they are replicated in close proximity, perhaps via replication fork collapse and repair, or when they recombine, especially at the repetitive sequences of pericentric heterochromatin [135]. Interestingly, inhibition of topoisomerase II reduces levels of homolog pairing in Drosophila cells, which may reflect different roles for topoisomerase in sister chromatid cohesion as versus homolog pairing ([93], see discussion within). Drosophila cells may also differ from other organisms in the timing of the resolution of catenations; for example, retention of catenations until mitosis, when perhaps they are resolved in response to spindle formation [131] or other mitosis-specific factors, might explain why cohesin knockdown did not perturb the cohesion of sister chromatids in G2. Indeed, the recent identification of ultrafine bridges in human cells demonstrates that catenations can remain until anaphase at certain regions (reviewed by [136]).

### Why have cohesin-independent cohesion?

If cohesin-independent connections exist between sister chromatids, why maintain another mechanism of cohesion in the form of the highly conserved and essential cohesin proteins? One explanation may be the requirement for unique connections between sister chromatids in order to ensure their segregation. Such connections may be provided by cohesin, whose establishment is coupled to DNA replication [18–20], while cohesin-independent mechanisms may contribute to genome organization in other ways. Secondly, cohesin-independent connections may allow cohesion to be maintained at chromosomal regions where cohesin protein is not always bound at a high density. This would enable cohesin binding to be spatially and temporally dynamic [42,137] and permit additional roles of cohesin in interphase, such as in the regulation of transcription and DNA repair [14,138]. Thus, cohesin-independent mechanisms contributing to cohesion, perhaps including the maintenance of catenations, may be especially important in cell types having a long G2 stage, such as the cells used in this study. Finally, having a diversity of cohesion mechanisms may allow for a more layered regulation of cohesion removal as cells enter mitosis [75]. In fact, in higher eukaryotes, cohesin proteins are removed from different parts of the chromosome at different times; while a small population of cohesin is retained at the centromeres and cleaved at anaphase [96,97,139], the bulk of cohesin on the chromosome arms is removed during prophase by Wapl and Pds5 [17,97,140–144]. Telomeric cohesion involves yet additional regulation [145–147]. Thus, cohesin-independent cohesion may constitute a further layer to be removed during the segregation of sister chromatids, the regulation of which may be useful in determining the order of segregation [51] or the length of the cell cycle. Since all these processes must be coordinated with the condensation of chromosomes prior to mitosis, perhaps it is not surprising that condensin proteins are involved in antagonizing cohesion (reviewed by [130]) or that cohesin and cohesin regulators play a role in condensation [11,46,148–150]. Overall, these observations and the work presented here indicate interesting interactions between different SMC complexes in the maintenance of interphase nuclear organization as well as the ways in which homologous DNA sequences interact with each other, whether between sister chromatids or between maternal and paternal homologs.

## Materials and Methods

### Cell culture and RNAi

Kc_167_, S2R+ and Clone 8 cells were cultured according to standard protocols (see www.flyrnai.org for more details). RNAi treatments were started in each case one day after the cells had been split as part of their regular passaging. RNAi treatments lasted for four days unless otherwise specified. For Kc_167_ and S2R+, cells were seeded at 0.5–0.8 million cells/mL with 15 μg of RNA per well in a 6-well plate or 5 μg of RNA per well in a 24-well plate (for double or triple knockdowns, these amounts were scaled accordingly, with the amount of RNA for each target being kept the same as in the single knockdowns). For Clone 8, cells were transfected with dsRNA using Effectene transfection reagent from Qiagen, with a GFP-expressing plasmid as a co-transfection marker. When using Effectene, the amount of dsRNA was reduced to 1.2 μg per well in a 24-well plate. dsRNA primers were designed using the SnapDragon tool for primer design (http://www.flyrnai.org/cgi-bin/RNAi_find_primers.pl) and synthesized by PCR amplification from genomic DNA followed by an *in vitro* transcription reaction using a MEGAscript T7 Transcription Kit (Thermo Fisher Scientific).

### qPCR

Quantitative PCR was used to assay efficiency of RNAi knockdowns according to standard techniques. Briefly, total RNA was isolated from cells using a Qiagen RNeasy Plus kit and then converted to cDNA using the Invitrogen SuperScript III First-Strand Synthesis System for RT-PCR. Primers for qPCR were designed using the Primer-BLAST website (http://www.ncbi.nlm.nih.gov/tools/primer-blast/). Reactions were set up using KAPA SYBR FAST qPCR kits and run on an Applied Biosystems 7300 Real-Time PCR System.

### Western blot

Cells were collected after four days of RNAi and their protein levels were analyzed by Western blot according to standard protocols. Blots were probed using a rabbit anti-Rad21 antibody (generous gift from Dr. Stefan Heidmann; used at 1:3000) to assay cohesin knockdown and a mouse anti-α-tubulin antibody (Sigma-Aldrich; 1:5000) to assay loading, followed by secondary antibodies conjugated to HRP (GE Healthcare Life Sciences), anti-rabbit (1:5000) and anti-mouse (1:10000). Blots were then stained using Pierce ECL Western Blotting Substrate (ThermoFisher Scientific). Band intensities were estimated using the gel analysis tools in ImageJ [151].

### FACS

Following RNAi treatment, cells were harvested, resuspended in ice cold 100% ethanol, allowed to warm up to 37°C and then stained using a PI/RNase Staining Buffer (BD Pharmingen). Cell populations were assayed based on DNA content to determine their cell cycle profile using a BD LSR II Analyzer.

### Immunofluorescence

Cells were plated onto slides at concentrations of 1–5 million cells/mL and allowed to adhere for 1–2 hours. The cells were then washed in PBS, and fixed in 4% paraformaldehyde for 5–10 minutes. The slides were then washed in PBS and used immediately or stored in PBS at 4°C. IF slides were washed in PBS-T (PBS with 0.1% Tween-20), blocked in 1% BSA/PBS-T for 30 minutes at room temperature, and incubated with primary antibody at 4°C overnight followed by three more PBS-T washes and incubation with secondary antibody either for 2 hours at room temperature or overnight at 4°C. Slides were then washed in PBS-T and mounted using Slowfade with DAPI (Thermo Fisher Scientific), followed by imaging.

Primary antibodies used: rabbit α-Rad21 (gift of Dr. Stefan Heidmann; 1:200), mouse α-cyclin B (Developmental Studies Hybridoma Bank; 1:100), rabbit α-pH3 S10 (Epitomics, 1:100). Secondary antibodies used (Jackson ImmunoResearch Laboratories): Cy3-conjugated anti-rabbit (1:165), 488-conjugated anti-mouse (1:100), Cy5-conjugated anti-mouse (1:20).

### FISH

Our protocol for fluorescence *in situ* hybridization has been previously published [64,91] and was adapted from standard protocols [85,152,153]. In brief, cells were fixed as above and then washed in PBS, 2x SSCT (0.3M sodium chloride, 0.03M sodium citrate, 0.1% Tween-20), and 50% formamide/2x SSCT. Slides were either used for FISH immediately or stored in 50% formamide/2x SSCT at 4°C. FISH slides were pre-denatured in 50% formamide/2x SSCT at 92°C for 2.5 minutes and then at 60°C for 20 minutes. FISH probes were added in a hybridization solution of 10% dextran sulphate/2x SSCT/50% formamide containing 10–20 pmol of probe per hybridization. The slides were then denatured by placing them on a heat block at 92°C for 2.5 minutes and allowed to hybridize overnight at room temperature for heterochromatic probes and at 37–42°C for euchromatic probes. Following hybridization, slides were washed in 2x SSCT at 60°C for 15 minutes, 2x SSCT at room temperature for 10 minutes, and 0.2x SSC at room temperature for 10 minutes before being mounted using Slowfade with DAPI (Thermo Fisher Scientific) and imaged. In cases where both IF and FISH were carried out, generally the two protocols were carried out in succession and the slides imaged afterwards. For some more sensitive antibodies, the cells were imaged following IF, then washed, used for FISH, and re-imaged, using software-assisted stage navigation to relocate the same fields.

Most euchromatic FISH probes used in this study were designed and generated using our published Oligopaints protocol [91], including 5A, 16E, 24D, 28B, 69C, 89B, 89E, and 100B, as well as the chromosome paints on 2R (41E-44C, 50D-53C, 58D-60E). One experiment at the 28B locus used a probe synthesized from a P1 plasmid (Berkeley Drosophila Genome Project) containing cloned Drosophila genomic DNA corresponding to chromosomal regions 28B1-28B2 (DS01529) and then labeled by nick translation/direct labeling (Vysis). Heterochromatic repeat regions were assayed using previously described FISH probe sequences [85,152] synthesized by Integrated DNA Technologies (IDT).

### Metaphase spreads

Metaphase cells were prepared using protocols adapted from published methods [154,155]. Cells were obtained from actively growing cultures without the use of drugs to increase mitotic index unless otherwise specified. In the case where microtubule inhibitors were used, colchicine was added to growing cells at a concentration of 30 μM for 2 hours prior to spread preparation. For all spreads, cells from 5 mL of culture were spun down, washed once in PBS, and then gradually resuspended in 10 mL 1% sodium citrate. The cells were incubated at room temperature for 30 minutes. We then added 1 mL of cold fixative (3:1 methanol: glacial acetic acid solution), spun down the cells, and washed three more times in 10 mL of the same fixative. Finally cells were resuspended in 100–500 μL fixative and dropped onto a glass slide under humidified conditions. The slide was allowed to dry and then washed in 70%, 90% and 100% ethanol successively, before being dried and imaged. For metaphase FISH, these slides were then denatured in 70% formamide/2X SSCT at 70°C for 90 seconds followed by washes in cold 70%, 90% and 100% ethanol. FISH probes were added and the cells were allowed to hybridize without any additional denaturing, followed by our standard FISH washes.

When scoring sister chromatid separation in metaphase spreads (without FISH), each spread was examined for the presence of single chromatids not attached to a sister along the entire chromosome arm. If unattached chromatids were visible, that metaphase was scored as having premature loss of cohesion, while if no unpaired chromatids were visible, it would be scored as having intact cohesion. When using FISH, the numbers of discrete FISH signals per metaphase were counted, as premature sister chromatid separation increases the number of FISH signals at pericentric loci from 1 to 2.

### Image acquisition and analysis

All images were obtained using an Olympus IX83 epifluorescence microscope with a 60x oil objective and the CellSens acquisition software. The raw TIFF files obtained were analyzed using custom-written MATLAB scripts (first described in [64] and subsequently adapted) for measuring different properties such as the number of FISH dots per nucleus, their area, and the intensity of IF signals. All uniquely identifiable foci of fluorescent signal (above background) were counted as FISH signals, regardless of the distance between them. The number of FISH signals and the area of FISH signals following cohesin knockdown was used as a measure of cohesion, defined as the close alignment of sister chromatids in interphase.

### Statistical analyses

When assaying the number of FISH signals in a nucleus, a whole population of cells was scored and each nucleus classified as either having one signal (homolog pairing as well as sister chromatid cohesion intact) or more than one signal (homologs have become unpaired or sisters have lost cohesion). The relative numbers of cells having one signal or more than one signal were then compared between different conditions using a two-tailed Fisher’s Exact Test. This type of analysis was also used when examining nuclei with higher numbers of FISH signals (i.e. nuclei were classified as having either up to four, or more than four, FISH signals per nucleus, etc.). When multiple trials of certain conditions were being compared, a two-tailed Student’s t-test was used to compare the percentages of nuclei with a single signal obtained under different conditions. A Student’s t-test was also used when comparing the distribution of FISH signal areas obtained when examining larger chromosomal regions. Finally, when examining the number of FISH signals in metaphase spreads, a Mann-Whitney U test was used to compare the different conditions.

## Supporting Information

S1 Fig Quantitative PCR confirmed efficient knockdown of cohesin subunits by RNAi. Results shown are for S2R+ cells following four days of RNAi. Relative mRNA levels were normalized to levels of rp49, a ribosomal gene, in each sample, and each sample was then normalized to levels in LacZ dsRNA-treated cells. Rad21 knockdown was confirmed to be more than 80% effective across multiple trials (left). Knockdowns of Smc1, Smc3 and SA were also found to be more than 79% effective in a single trial (right).
(TIF)

S2 Fig Knockdown of Rad21 is efficient in Kc_167_ cells and disrupts metaphase cohesion. (A) Western blot showing that knockdown is efficient after four days of RNAi. (B) Immunofluorescence for Rad21 confirms knockdown in individual cells. (C) Metaphase spreads show that Rad21 knockdown causes premature sister chromatid separation, with a significant increase in the number of FISH signals targeting AACAC and dodeca (P<0.0001) but not 359 (P = 0.1556). n≥29 mitotic nuclei per knockdown, differences between untreated cells and Rad21 RNAi treated cells were calculated by Mann-Whitney U test.
(TIF)

S3 Fig Efficiency of Rad21 knockdown in S2R+ cells is estimated to be 88–89%. Western blots prepared from cells treated for four days with either lacZ dsRNA or Rad21 dsRNA, using (A) various concentrations of anti-Rad21 antibody for probing, and (B) various exposure times when imaging the blots. At high antibody concentrations and exposure times, residual cohesin can be observed. Quantification of band intensities estimated the amount of cohesin remaining to be 11–12% of control levels, indicating a knockdown efficiency of 88–89%. While a more accurate estimate of knockdown efficiency would be obtained following an antibody titration, our results, especially when combined with the immunofluorescence and qPCR data, indicate that the knockdown of Rad21 in these cells is fairly efficient.
(TIF)

S4 Fig Knockdowns of multiple cohesin subunits, and longer RNAi treatments, did not disrupt interphase alignment of sister chromatids and homologous chromosomes. (A) Graphs showing the percentages of nuclei with a single FISH signal at several heterochromatic and euchromatic loci following knockdowns of different cohesin proteins Rad21, Smc1, Smc3 and SA in various combinations, in both S2R+ and Kc_167_ cells. (B) Graphs showing the percentages of nuclei with a single FISH signal after Rad21 was knocked down for periods longer than our standard 4 day RNAi treatment, in both S2R+ and Kc_167_ cells. (For all graphs, shown are percentages from single trials, n≥290 nuclei per knockdown.)
(TIF)

S5 Fig Knockdowns of cohesin subunits cause premature loss of cohesion in mitosis. Metaphase spreads obtained from Kc_167_ cells (tetraploid) after (A) no dsRNA treatment, (B) Rad21 RNAi, (C) Smc1 RNAi and (D) Smc3 RNAi. Spreads were prepared following four days of RNAi without use of any drugs to increase mitotic index. Rad21 knockdown caused a more severe loss-of-cohesion phenotype as compared to knockdowns of Smc1 and Smc3.
(TIF)

S6 Fig Cells continue to cycle following Rad21 knockdown while exhibiting metaphase cohesion defects. (A) Growth curves of Kc_167_ cells with no dsRNA (dark blue) and treated with Rad21 dsRNA (light blue). dsRNA was added at day zero and cell count was assayed every day for 6 days. Rad21 knockdown caused a slight cell cycle delay compared to untreated cells. (B) FACS profiles of S2R+ cells subjected to 5 days of Rad21 knockdown and stained with propidium iodide to assay DNA content. Rad21 RNAi caused a slight enrichment for G2 cells compared to cells treated with LacZ RNAi. (C) Timecourse showing gradual onset of the premature loss of cohesion phenotype in response to Rad21 RNAi in Kc_167_ cells. dsRNA was added at day zero and metaphase spreads were prepared each day for 6 days from untreated and Rad21 RNAi-treated cells.
(TIF)

S7 Fig Efficiency of RNAi knockdown in Clone 8 cells must be assayed on a cell-by-cell basis. Shown is immunofluorescence for Rad21 protein (scale bar = 10 μm). Unlike in S2R+ (Fig 2B) or Kc167 cells (S2B Fig), RNAi in Clone 8 cells is not 100% efficient when assayed at a population level. Immunofluorescence after Rad21 RNAi demonstrates that some cells are depleted for Rad21 and only show background levels of fluorescence, while other cells show fluorescence intensities that are comparable to that of control cells. This variability is the result of a limited transfection efficiency, such that only 30–40% of the cells take up the dsRNA. Therefore, for the data shown in Fig 4, only cells that were positive for GFP (transfection marker), negative for Rad21, and positive for Cyclin B (G2 marker) were scored for their FISH phenotypes.
(TIF)

S8 Fig Knockdowns of genes in double and triple RNAi treatments are as efficient as single knockdowns. Quantitative PCR results are shown for S2R+ cells (processed in the same way as S1 Fig). For single knockdowns, 5 μg of dsRNA was used while for multiple knockdowns, 5 μg of the dsRNA of each species was used (i.e. 10 μg total in double knockdowns, 15 μg in triple knockdowns) and the controls used were untreated cells.
(TIF)

S9 Fig Double knockdown of Rad21 and Slmb produces more FISH signals than does knockdown of Slmb alone. Data shown is the same as in Fig 5B and 5C, but nuclei are sorted by the actual number of FISH signals per nucleus (rather than the proportion having more than three signals, etc.). Frequency was calculated by dividing the number of nuclei having a specific number of FISH signals by the total amount of nuclei scored. Results are the mean of at least 6 independent trials, error bars = SD, n≥100 nuclei per knockdown per trial.
(TIF)

S10 Fig Knockdown of Rad21 and Slmb did not cause more aneuploidy than did knockdown of either gene alone. Metaphase spreads were prepared from S2R+ cells following four days of RNAi (results are from a single trial; n = 30 mitotic nuclei per knockdown). Knockdowns involving Rad21 double the number of FISH signals observed at AACAC and dodeca because of sister chromatid separation (for discussion of 359, see main text; P-values from Mann-Whitney U-test comparing LacZ and Rad21 RNAi are <0.0001 for AACAC and dodeca, P = 0.0568 for 359). In the double knockdown of Rad21 and Slmb, this doubling in the number of FISH signals was also observed, but the overall number of chromatid pairs was not significantly increased compared to knockdowns of either Rad21 or Slmb alone (P-values from Mann-Whitney U-test comparing Rad21 RNAi and Rad21+Slmb RNAi are 0.7864, 0.5132, and 0.1652 for 359, AACAC, and dodeca, respectively).
(TIF)

S11 Fig Knockdown of Rad21 and Slmb increases the number of FISH signals at certain euchromatic loci. Results shown are for S2R+ cells following four days of RNAi. The number of FISH signals described as “extra” depends on the copy number of the chromosome being examined. For FISH targets on the X chromosome (16E), chromosome 2 (24D and 28B), and chromosome 3 (69C and 100B), respectively, nuclei with greater than or equal to 3, 4 or 5 signals were classified as having extra FISH signals (see main text for more details). Shown are the percentages from single trials (n≥300 per knockdown). While the increases observed were modest compared to those seen at heterochromatic loci, significant increases were seen at 3 out of 5 loci examined (significance calculated by Fisher’s exact test, *, P<0.05 for difference between Slmb knockdown and Rad21+Slmb double knockdown; for 24D, 100B, 16E, 69C and 28B, respectively, P = 0.0150, P = 0.0447, P = 0.0026, P = 0.1382, P = 0.0991).
(TIF)

S12 Fig Slmb knockdown, alone or in combination with Rad21 and/or Cap-H2, leads to fewer G2 cells. (A) Representative control cells stained with antibodies against cyclin B. Cells that would be identified as “G2” are outlined with a dashed line (scale bar = 10 μm). Mitotic cells also express cyclin B, but were excluded on the basis of DAPI morphology. (B) Quantification of the percentage of G2 cells observed after four days of RNAi knockdowns in S2R+ cells (n≥100 cells per knockdown).
(TIF)

S13 Fig In mitosis, condensin I plays more of a role in antagonizing sister chromatid cohesion than does condensin II. (A) Rad21 knockdown leads to an increase in the number of FISH signals in mitotic nuclei, which are identified by staining for phosphorylated histone H3 (pH3). (B) Rad21 knockdown also leads to abnormal morphologies of mitotic nuclei, including multi-lobed structures with breaks in the mass of chromosomes formed at mitosis (dotted line; DAPI perimeter). (C) Depletion of condensin protein Smc2, present in both condensin I and II, partially rescues defects observed after Rad21 knockdown; nuclear morphology resembles that of control cells, consistent with published results [29,109]. (D) Quantification of results for experiments illustrated in (B) and (C). Mitotic nuclei were classified as either having (Discontinuous) or not having (Continuous) discontinuities in structure, as revealed by pH3 staining. Results are shown for Rad21 knockdown alone and in combination with that of Smc2 (condensin I and II), Barren (condensin I only), and Cap-H2 (condensin II only). Significant rescues were observed when condensin I proteins were knocked down in addition to Rad21, but not with condensin II. This finding suggests that whatever function is played by condensin II in antagonizing sister chromatid cohesion in interphase is at least partially redundant with that played by condensin I in mitosis. Means represent three independent trials in S2R+ cells; error bars = SD; n≥53 mitotic nuclei per knockdown per trial; significance calculated for each trial using Fisher’s exact test (*, P<0.0001 in both trials; ~, P = 0.1914 in one trial and P = 0.0111 in another). Mitotic index for each knockdown, determined as the percentage of pH3-positive nuclei, is shown beneath the graph (n≥850 per genotype).
(TIF)

S14 Fig Schematic showing ways in which transvection between sister chromatids might be synergistic or mutually inhibitory with that between homologs. (TIF)

S1 Table Separation of sister chromatids following Rad21 RNAi is not dependent on microtubule polymerization. Metaphase spreads were scored as either having intact cohesion (all visible chromatids were attached to a sister chromatid) or not (unattached chromatids were visible, indicative of premature loss of sister chromatid cohesion). The addition of colchicine does not significantly affect the percentage of cells with intact cohesion following Rad21 knockdown (P = 0.1570) or in cells untreated with dsRNA (P = 0.3553). Even after colchicine treatment, the percentage of cells with intact cohesion is significantly reduced following Rad21 knockdown compared to cells untreated with dsRNA (P<0.0001).
(TIF)
