# Supplementary figures and images for: Investigating the Interplay between Sister Chromatid Cohesion and Homolog Pairing in Drosophila Nuclei
Source: PLoS Genet. 2016 Aug 19;12(8):e1006169. doi: 10.1371/journal.pgen.1006169 (PMC4991795; doi:10.1371/journal.pgen.1006169)

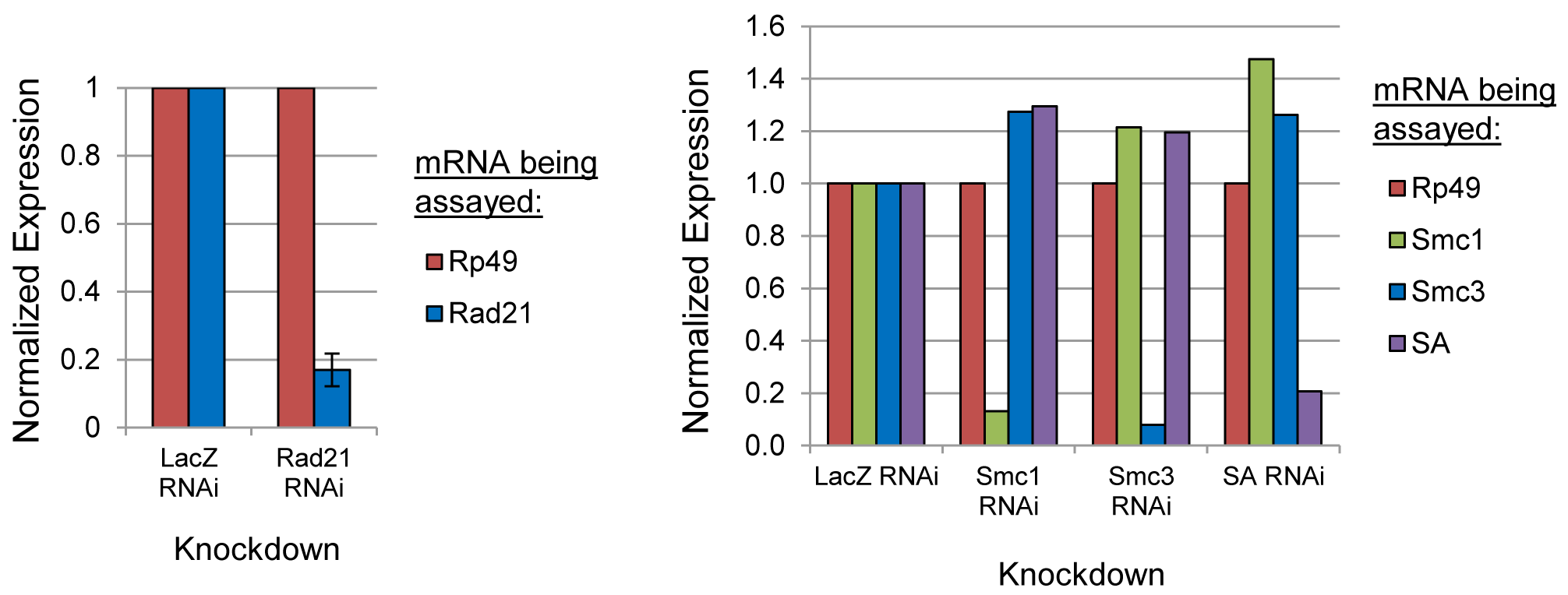

Supplement: S1 Fig — Results shown are for S2R+ cells following four days of RNAi. Relative mRNA levels were normalized to levels of rp49, a ribosomal gene, in each sample, and each sample was then normalized to levels in LacZ dsRNA-treated cells. Rad21 knockdown was confirmed to be more than 80% effective across multiple trials (left). Knockdowns of Smc1, Smc3 and SA were also found to be more than 79% effective in a single trial (right). (TIF) [file pgen.1006169.s001.tif]

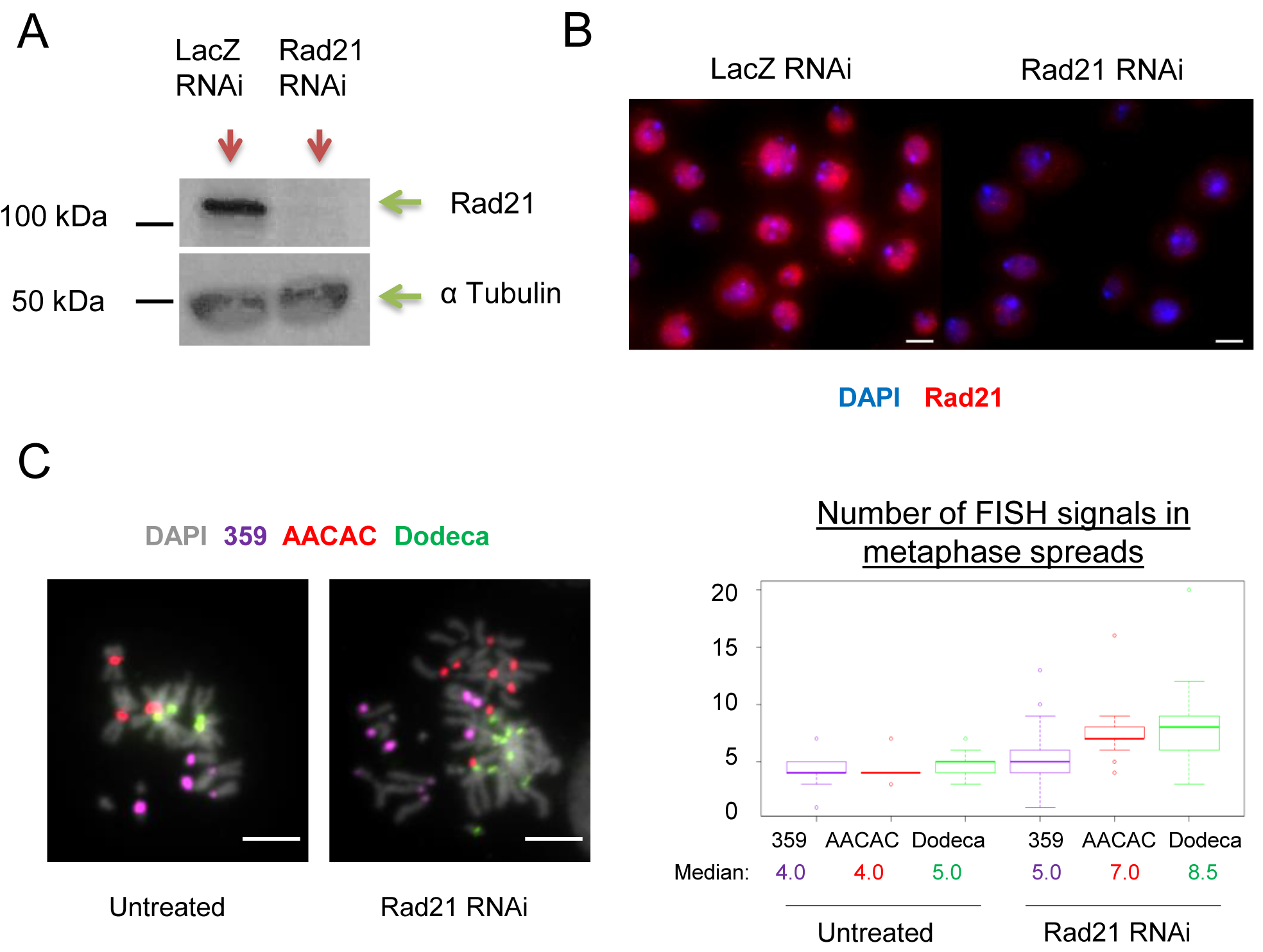

Supplement: S2 Fig — (A) Western blot showing that knockdown is efficient after four days of RNAi. (B) Immunofluorescence for Rad21 confirms knockdown in individual cells. (C) Metaphase spreads show that Rad21 knockdown causes premature sister chromatid separation, with a significant increase in the number of FISH signals targeting AACAC and dodeca (P<0.0001) but not 359 (P = 0.1556). n≥29 mitotic nuclei per knockdown, differences between untreated cells and Rad21 RNAi treated cells were calculated by Mann-Whitney U test. (TIF) [file pgen.1006169.s002.tif]

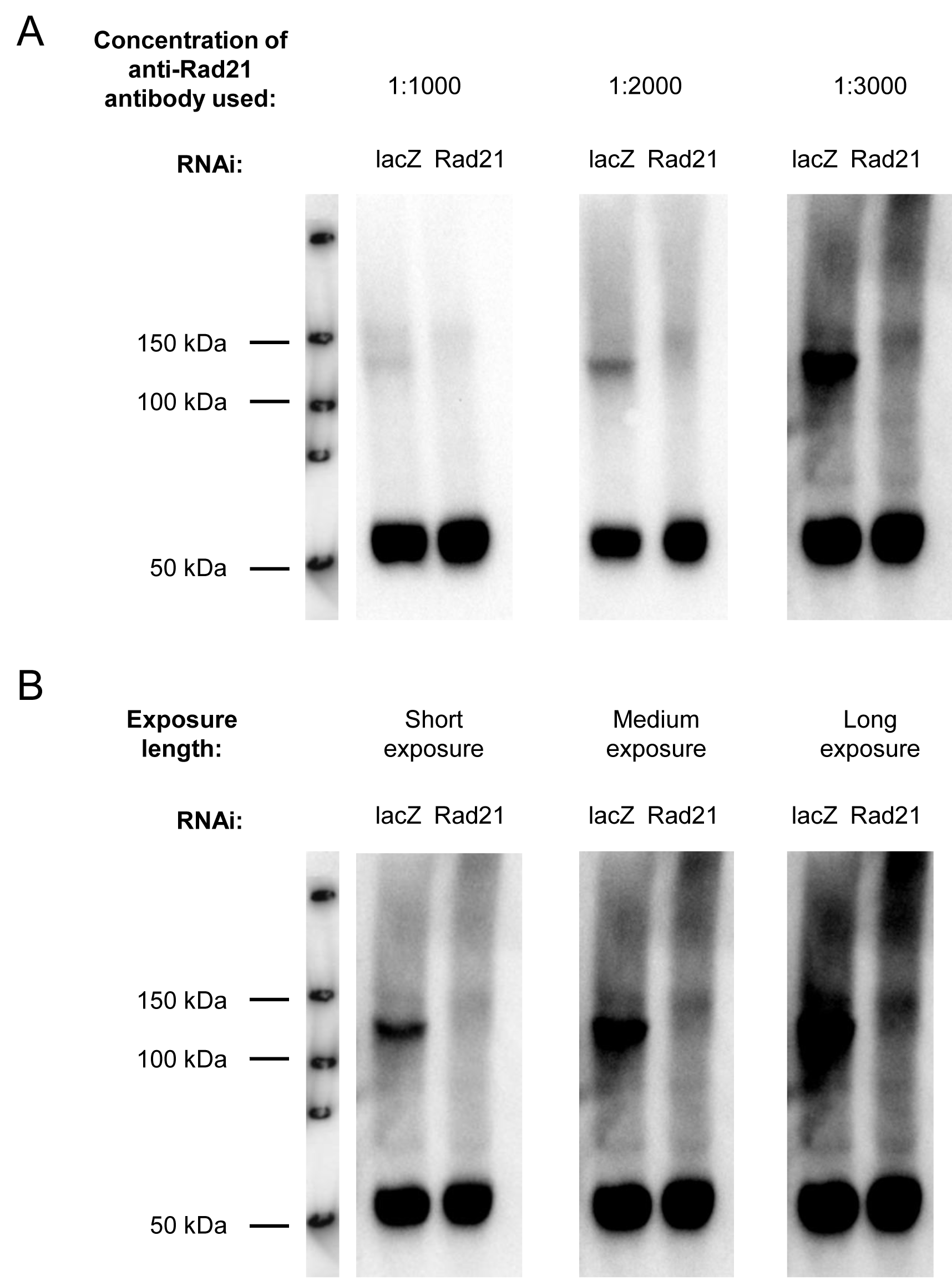

Supplement: S3 Fig — Western blots prepared from cells treated for four days with either lacZ dsRNA or Rad21 dsRNA, using (A) various concentrations of anti-Rad21 antibody for probing, and (B) various exposure times when imaging the blots. At high antibody concentrations and exposure times, residual cohesin can be observed. Quantification of band intensities estimated the amount of cohesin remaining to be 11–12% of control levels, indicating a knockdown efficiency of 88–89%. While a more accurate estimate of knockdown efficiency would be obtained following an antibody titration, our results, especially when combined with the immunofluorescence and qPCR data, indicate that the knockdown of Rad21 in these cells is fairly efficient. (TIF) [file pgen.1006169.s003.tif]

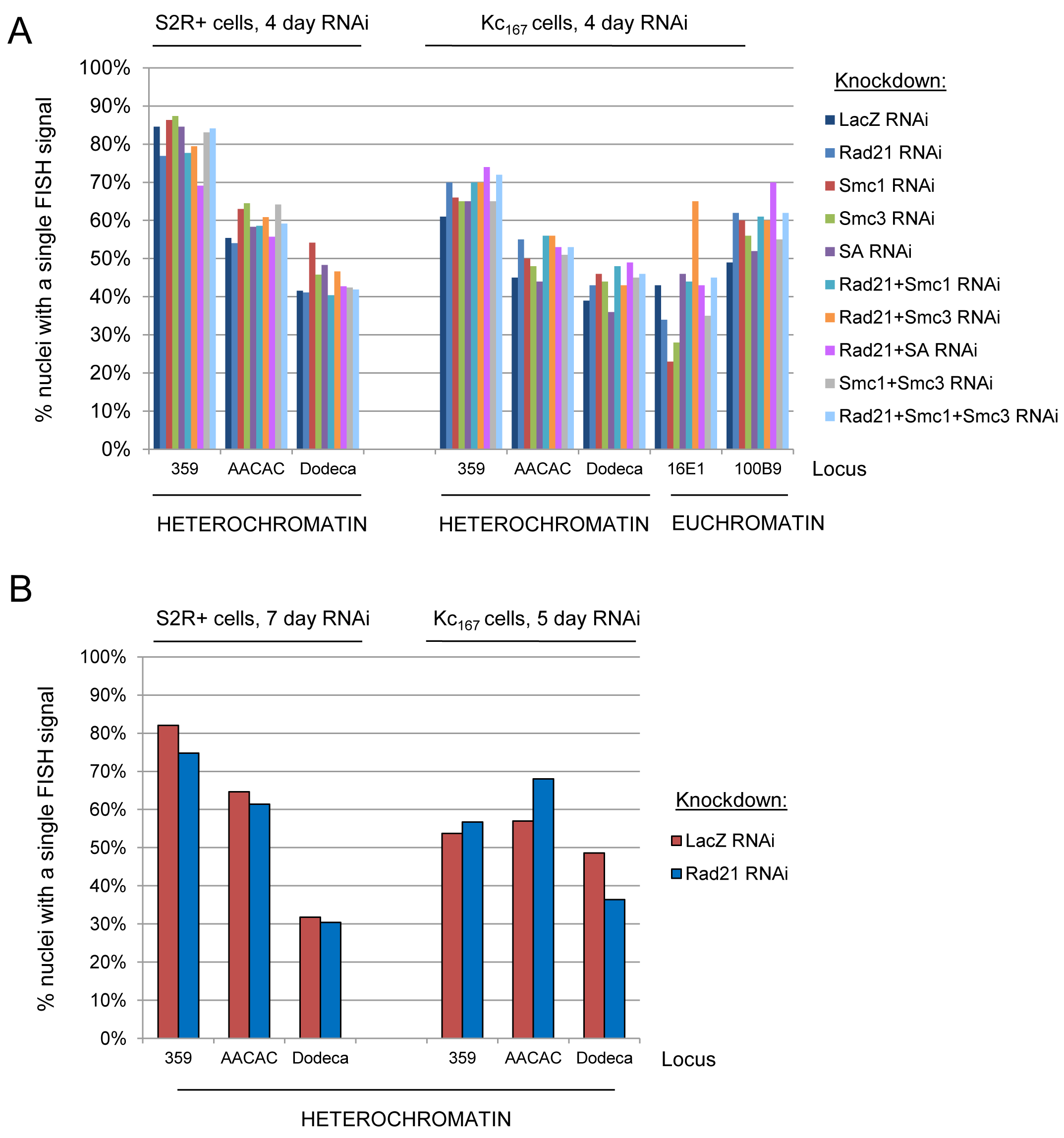

Supplement: S4 Fig — (A) Graphs showing the percentages of nuclei with a single FISH signal at several heterochromatic and euchromatic loci following knockdowns of different cohesin proteins Rad21, Smc1, Smc3 and SA in various combinations, in both S2R+ and Kc167 cells. (B) Graphs showing the percentages of nuclei with a single FISH signal after Rad21 was knocked down for periods longer than our standard 4 day RNAi treatment, in both S2R+ and Kc167 cells. (For all graphs, shown are percentages from single trials, n≥290 nuclei per knockdown.) (TIF) [file pgen.1006169.s004.tif]

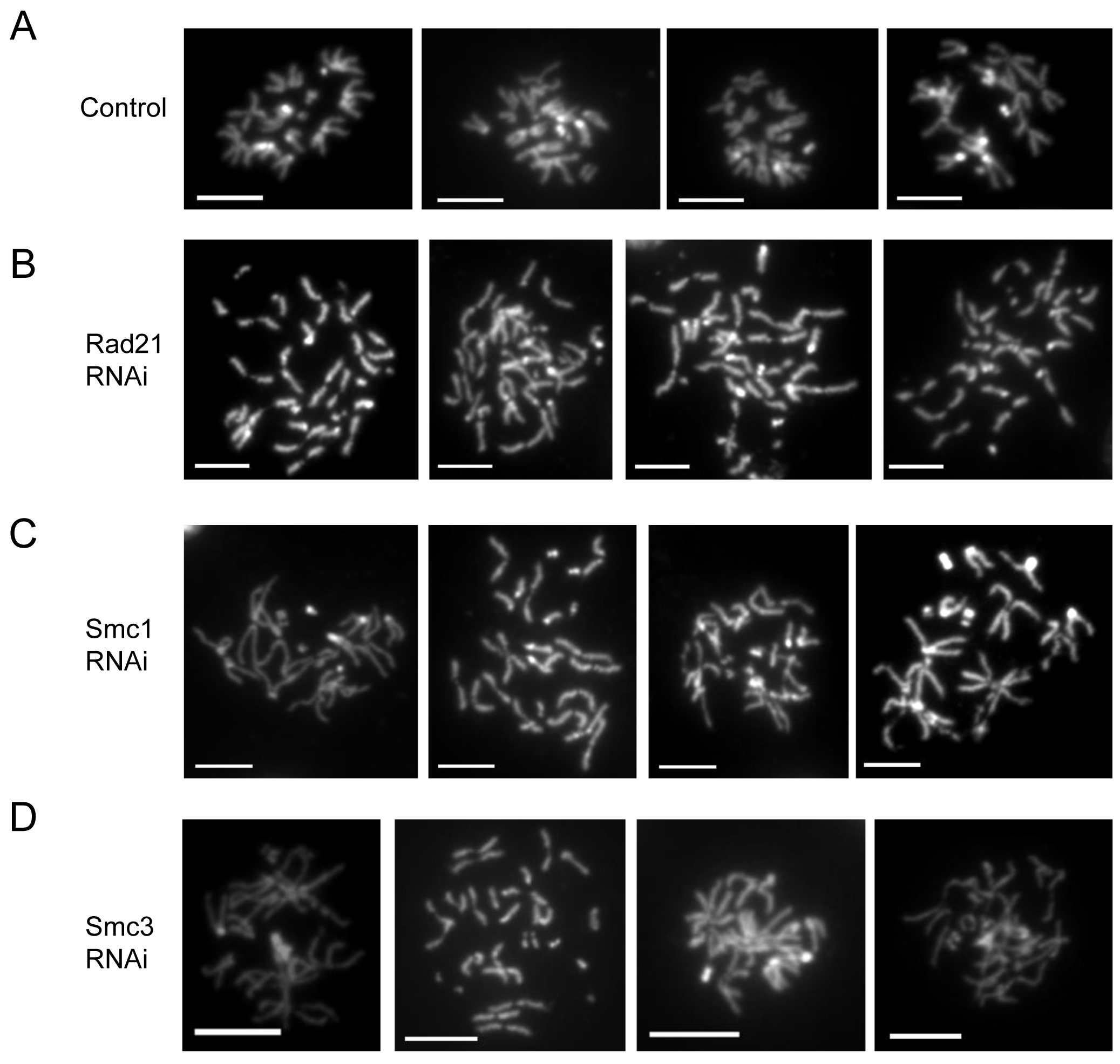

Supplement: S5 Fig — Metaphase spreads obtained from Kc167 cells (tetraploid) after (A) no dsRNA treatment, (B) Rad21 RNAi, (C) Smc1 RNAi and (D) Smc3 RNAi. Spreads were prepared following four days of RNAi without use of any drugs to increase mitotic index. Rad21 knockdown caused a more severe loss-of-cohesion phenotype as compared to knockdowns of Smc1 and Smc3. (TIF) [file pgen.1006169.s005.tif]

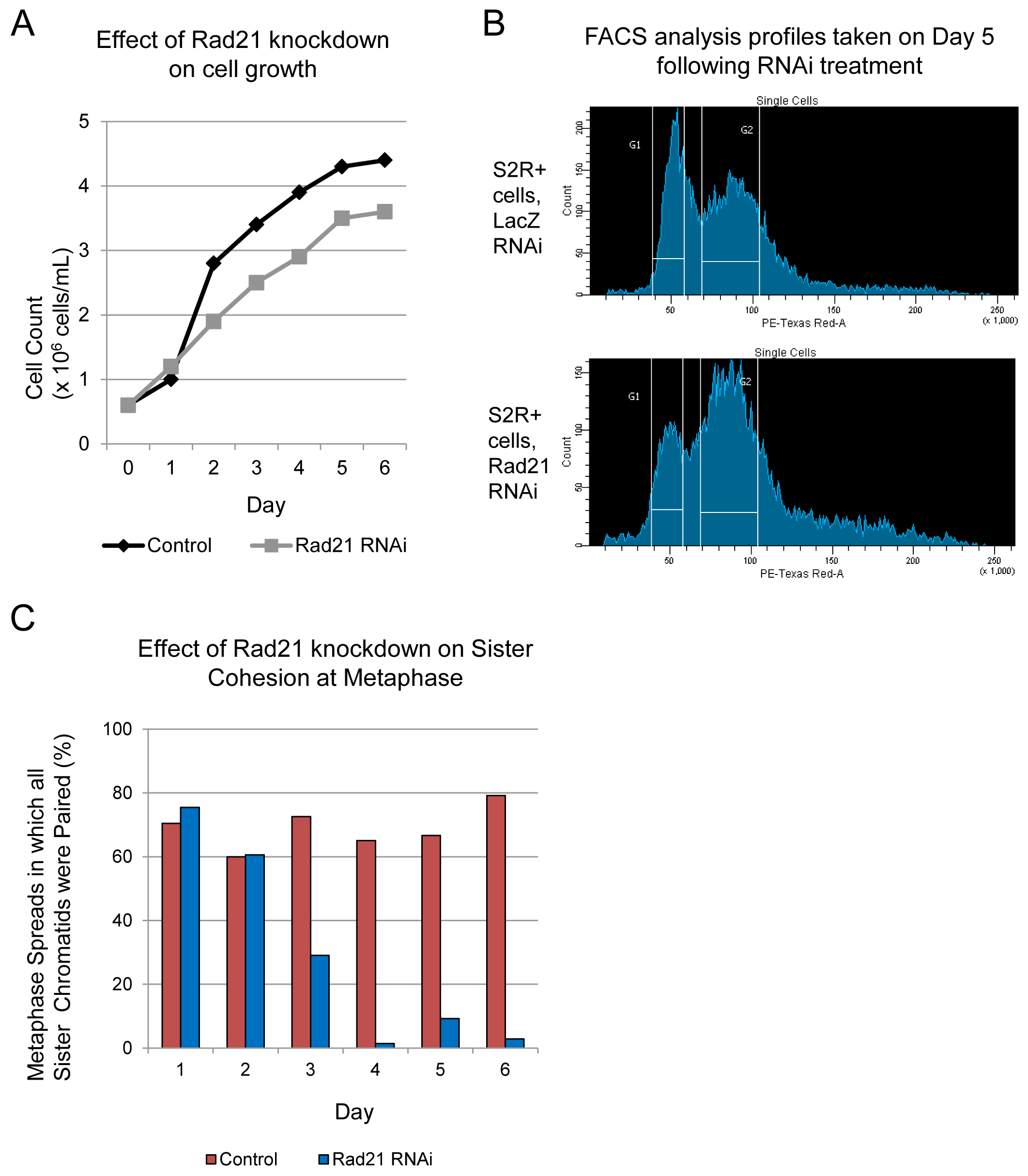

Supplement: S6 Fig — (A) Growth curves of Kc167 cells with no dsRNA (dark blue) and treated with Rad21 dsRNA (light blue). dsRNA was added at day zero and cell count was assayed every day for 6 days. Rad21 knockdown caused a slight cell cycle delay compared to untreated cells. (B) FACS profiles of S2R+ cells subjected to 5 days of Rad21 knockdown and stained with propidium iodide to assay DNA content. Rad21 RNAi caused a slight enrichment for G2 cells compared to cells treated with LacZ RNAi. (C) Timecourse showing gradual onset of the premature loss of cohesion phenotype in response to Rad21 RNAi in Kc167 cells. dsRNA was added at day zero and metaphase spreads were prepared each day for 6 days from untreated and Rad21 RNAi-treated cells. (TIF) [file pgen.1006169.s006.tif]

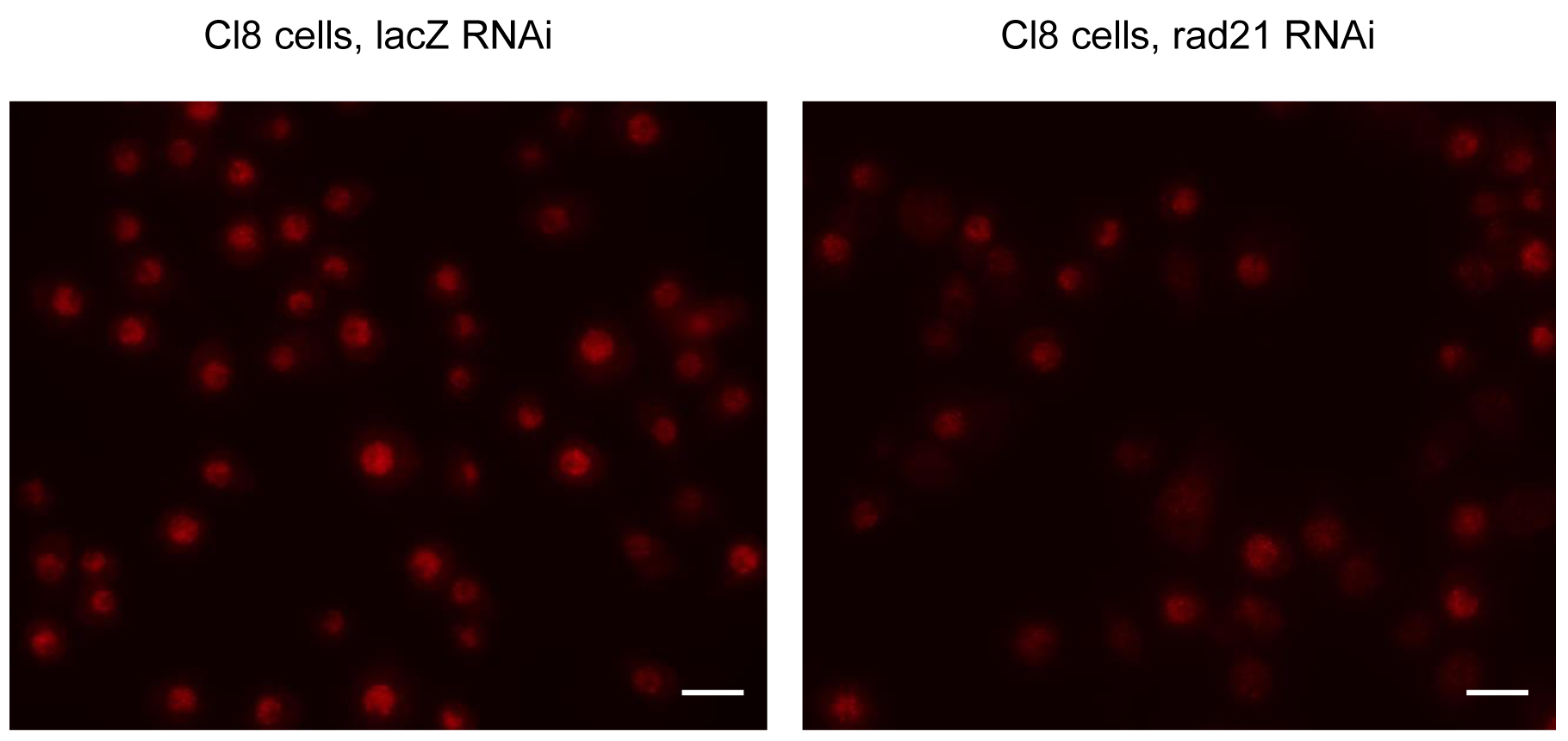

Supplement: S7 Fig — Shown is immunofluorescence for Rad21 protein (scale bar = 10 μm). Unlike in S2R+ (Fig 2B) or Kc167 cells (S2B Fig), RNAi in Clone 8 cells is not 100% efficient when assayed at a population level. Immunofluorescence after Rad21 RNAi demonstrates that some cells are depleted for Rad21 and only show background levels of fluorescence, while other cells show fluorescence intensities that are comparable to that of control cells. This variability is the result of a limited transfection efficiency, such that only 30–40% of the cells take up the dsRNA. Therefore, for the data shown in Fig 4, only cells that were positive for GFP (transfection marker), negative for Rad21, and positive for Cyclin B (G2 marker) were scored for their FISH phenotypes. (TIF) [file pgen.1006169.s007.tif]

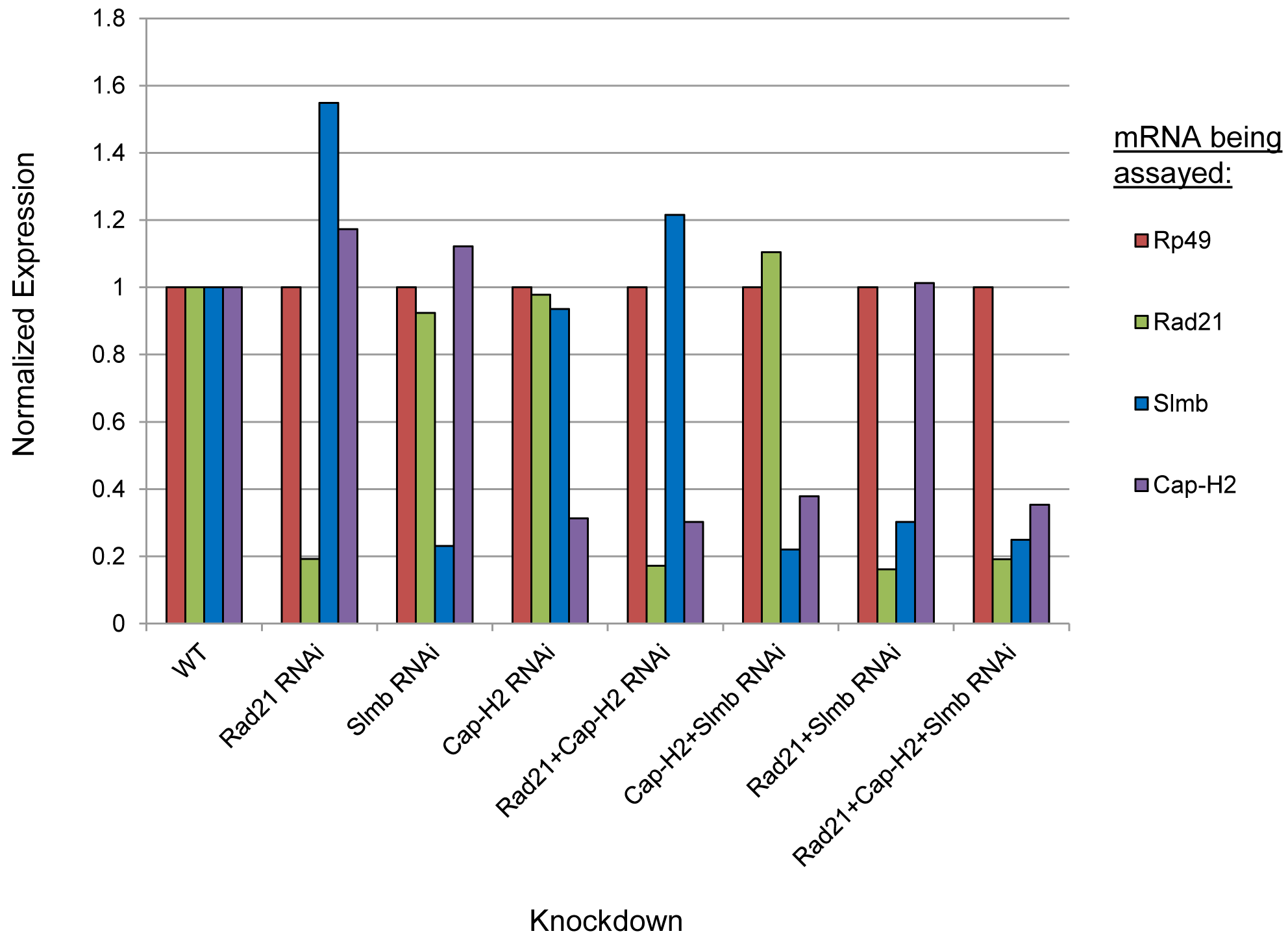

Supplement: S8 Fig — Quantitative PCR results are shown for S2R+ cells (processed in the same way as S1 Fig). For single knockdowns, 5 μg of dsRNA was used while for multiple knockdowns, 5 μg of the dsRNA of each species was used (i.e. 10 μg total in double knockdowns, 15 μg in triple knockdowns) and the controls used were untreated cells. (TIF) [file pgen.1006169.s008.tif]

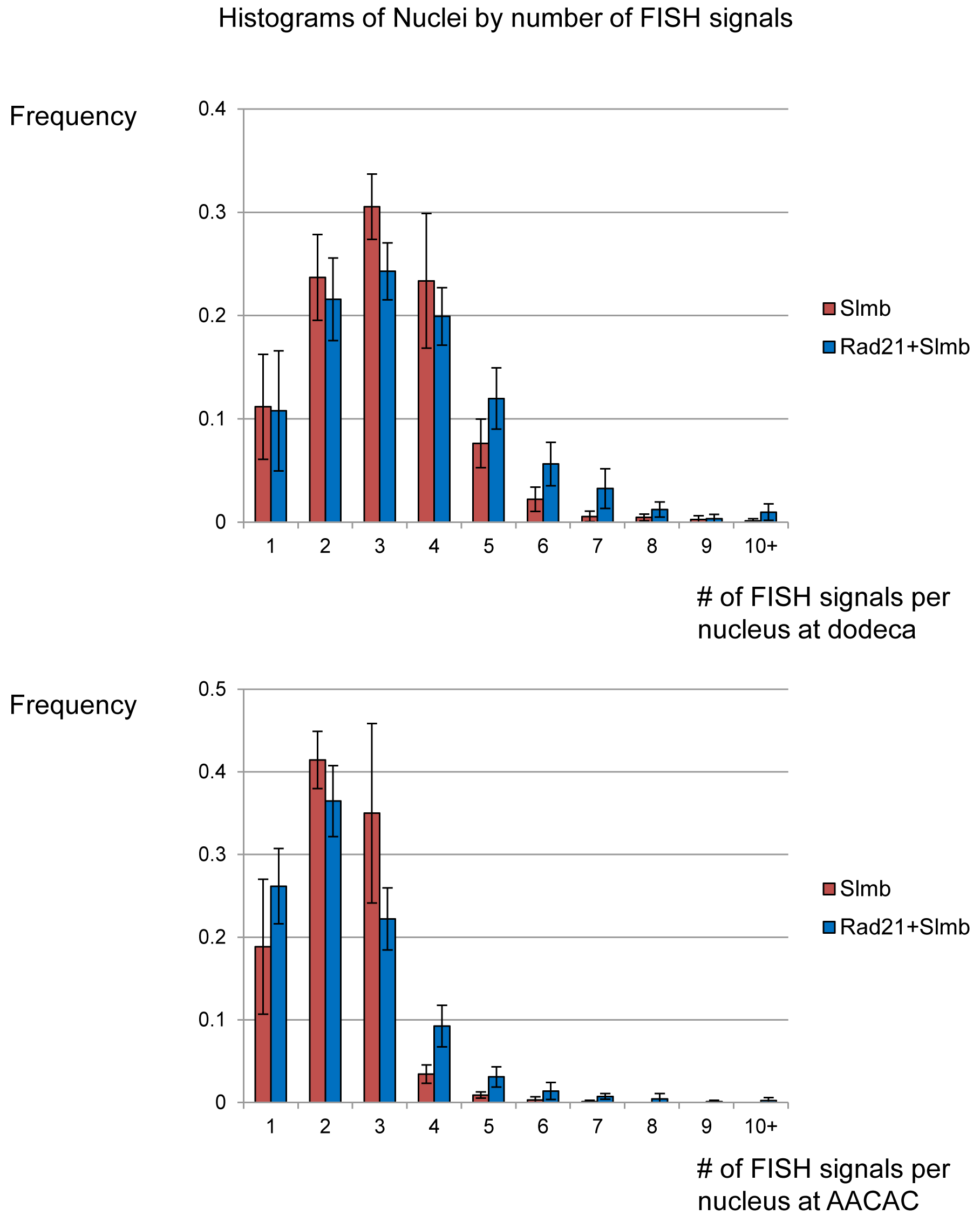

Supplement: S9 Fig — Data shown is the same as in Fig 5B and 5C, but nuclei are sorted by the actual number of FISH signals per nucleus (rather than the proportion having more than three signals, etc.). Frequency was calculated by dividing the number of nuclei having a specific number of FISH signals by the total amount of nuclei scored. Results are the mean of at least 6 independent trials, error bars = SD, n≥100 nuclei per knockdown per trial. (TIF) [file pgen.1006169.s009.tif]

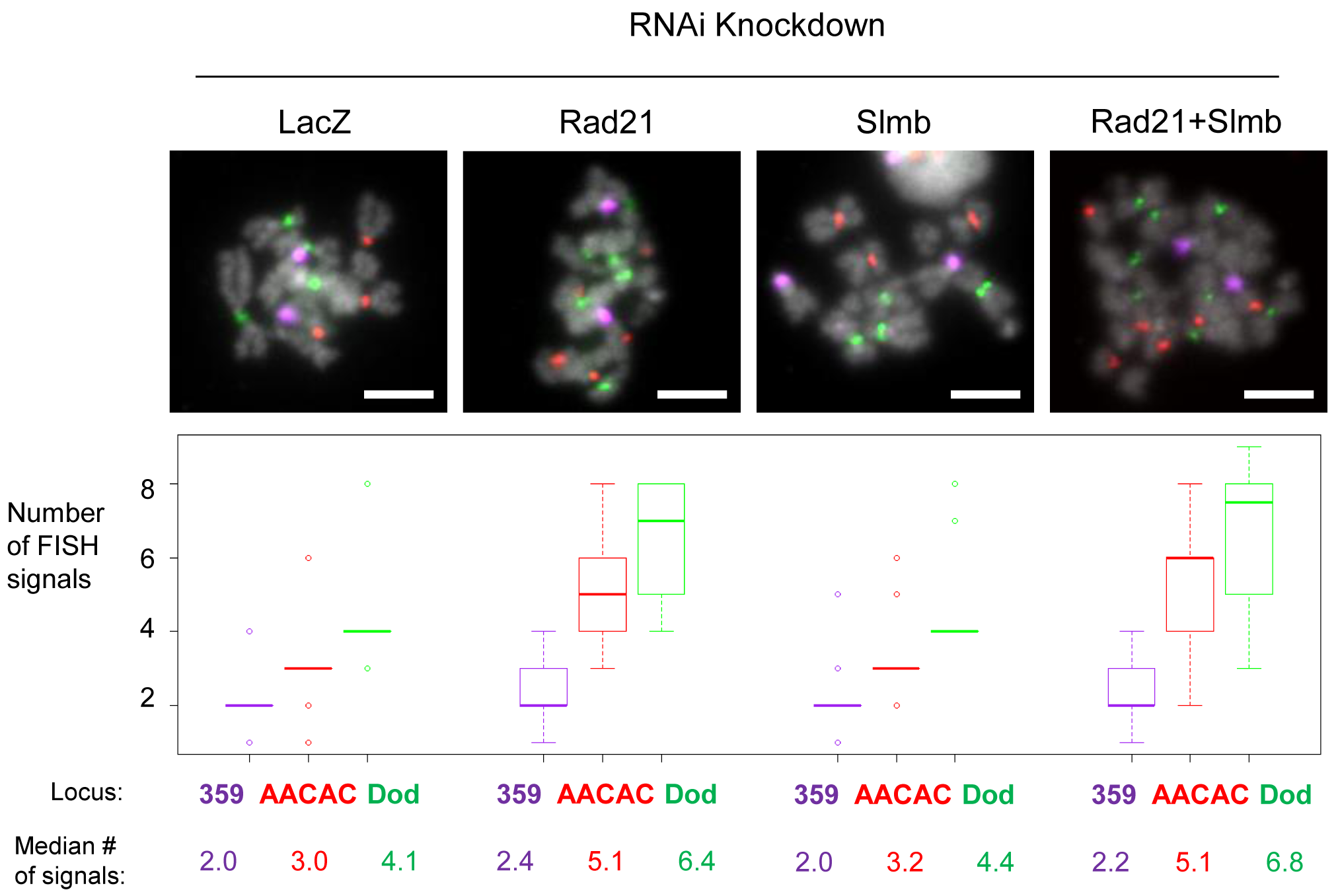

Supplement: S10 Fig — Metaphase spreads were prepared from S2R+ cells following four days of RNAi (results are from a single trial; n = 30 mitotic nuclei per knockdown). Knockdowns involving Rad21 double the number of FISH signals observed at AACAC and dodeca because of sister chromatid separation (for discussion of 359, see main text; P-values from Mann-Whitney U-test comparing LacZ and Rad21 RNAi are <0.0001 for AACAC and dodeca, P = 0.0568 for 359). In the double knockdown of Rad21 and Slmb, this doubling in the number of FISH signals was also observed, but the overall number of chromatid pairs was not significantly increased compared to knockdowns of either Rad21 or Slmb alone (P-values from Mann-Whitney U-test comparing Rad21 RNAi and Rad21+Slmb RNAi are 0.7864, 0.5132, and 0.1652 for 359, AACAC, and dodeca, respectively). (TIF) [file pgen.1006169.s010.tif]

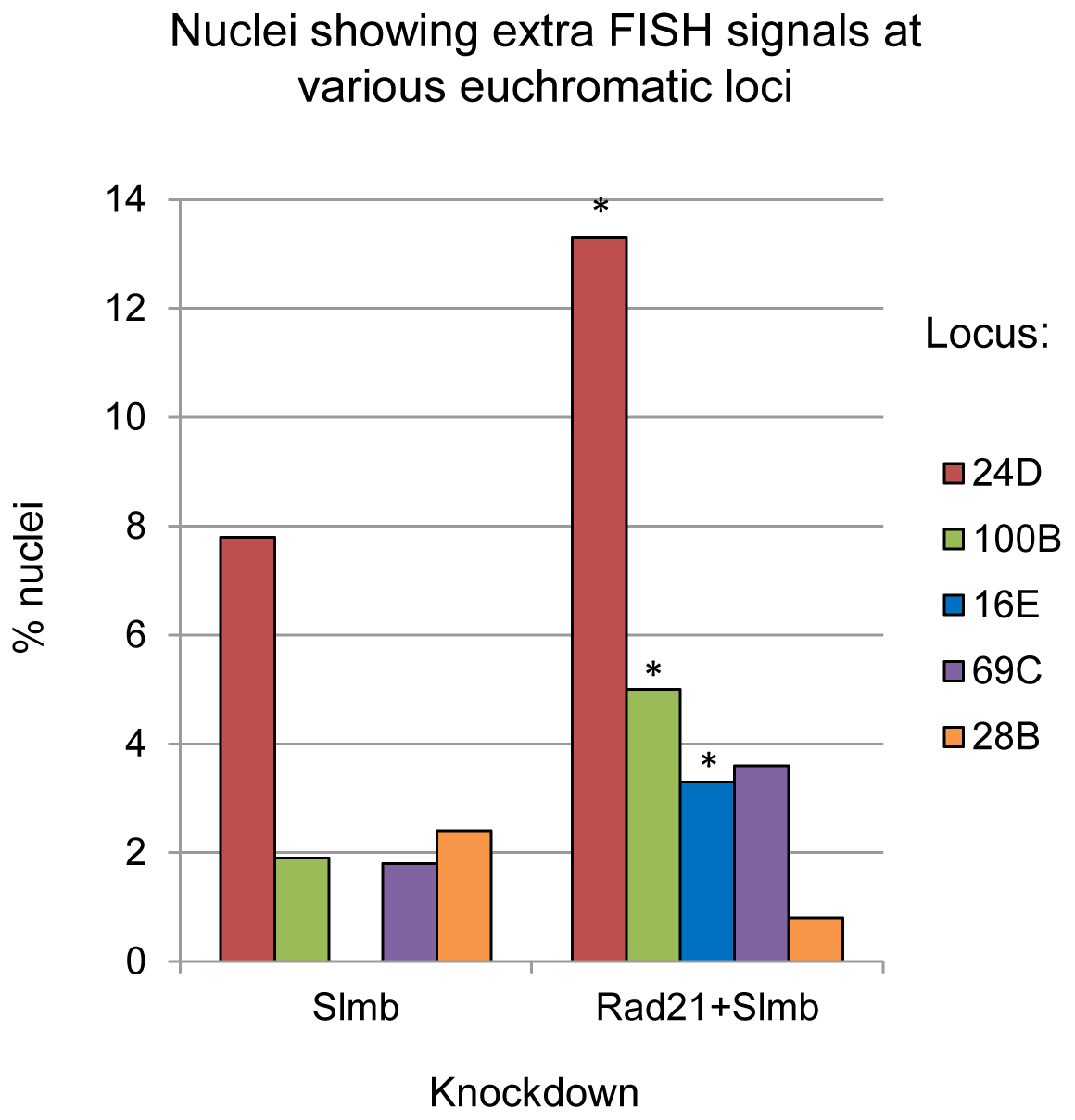

Supplement: S11 Fig — Results shown are for S2R+ cells following four days of RNAi. The number of FISH signals described as “extra” depends on the copy number of the chromosome being examined. For FISH targets on the X chromosome (16E), chromosome 2 (24D and 28B), and chromosome 3 (69C and 100B), respectively, nuclei with greater than or equal to 3, 4 or 5 signals were classified as having extra FISH signals (see main text for more details). Shown are the percentages from single trials (n≥300 per knockdown). While the increases observed were modest compared to those seen at heterochromatic loci, significant increases were seen at 3 out of 5 loci examined (significance calculated by Fisher’s exact test, *, P<0.05 for difference between Slmb knockdown and Rad21+Slmb double knockdown; for 24D, 100B, 16E, 69C and 28B, respectively, P = 0.0150, P = 0.0447, P = 0.0026, P = 0.1382, P = 0.0991). (TIF) [file pgen.1006169.s011.tif]

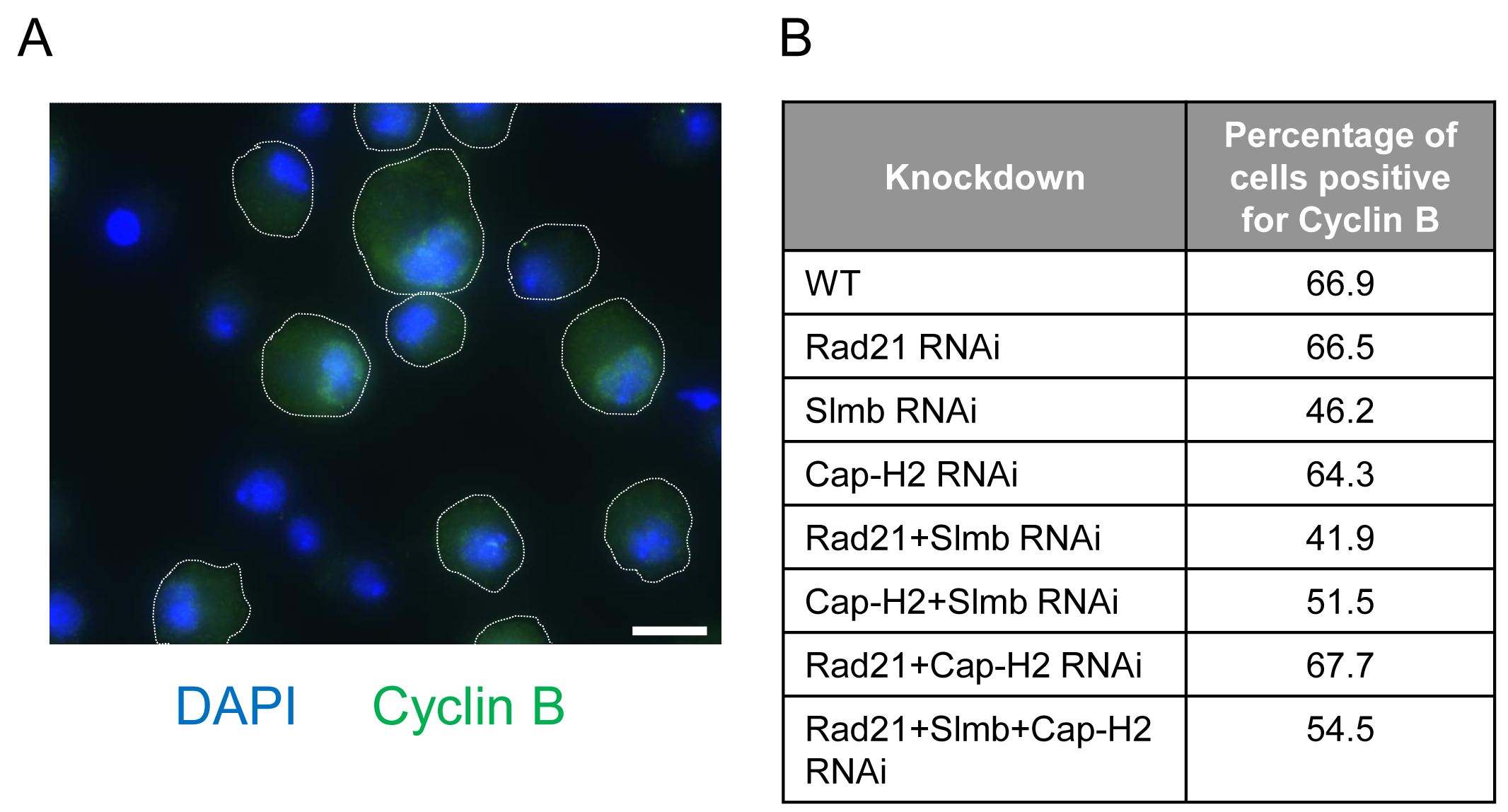

Supplement: S12 Fig — (A) Representative control cells stained with antibodies against cyclin B. Cells that would be identified as “G2” are outlined with a dashed line (scale bar = 10 μm). Mitotic cells also express cyclin B, but were excluded on the basis of DAPI morphology. (B) Quantification of the percentage of G2 cells observed after four days of RNAi knockdowns in S2R+ cells (n≥100 cells per knockdown). (TIF) [file pgen.1006169.s012.tif]

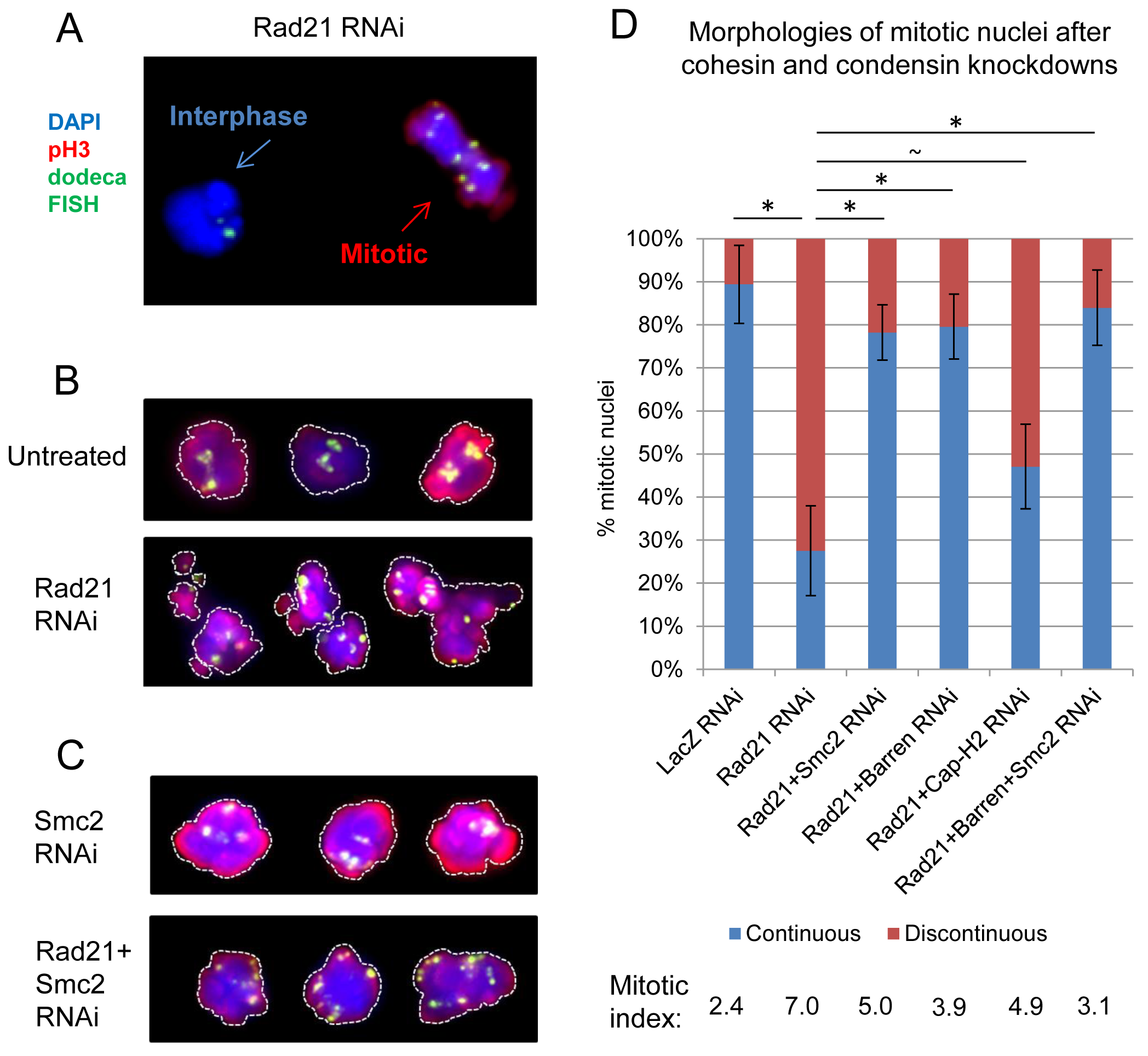

Supplement: S13 Fig — (A) Rad21 knockdown leads to an increase in the number of FISH signals in mitotic nuclei, which are identified by staining for phosphorylated histone H3 (pH3). (B) Rad21 knockdown also leads to abnormal morphologies of mitotic nuclei, including multi-lobed structures with breaks in the mass of chromosomes formed at mitosis (dotted line; DAPI perimeter). (C) Depletion of condensin protein Smc2, present in both condensin I and II, partially rescues defects observed after Rad21 knockdown; nuclear morphology resembles that of control cells, consistent with published results [29,109]. (D) Quantification of results for experiments illustrated in (B) and (C). Mitotic nuclei were classified as either having (Discontinuous) or not having (Continuous) discontinuities in structure, as revealed by pH3 staining. Results are shown for Rad21 knockdown alone and in combination with that of Smc2 (condensin I and II), Barren (condensin I only), and Cap-H2 (condensin II only). Significant rescues were observed when condensin I proteins were knocked down in addition to Rad21, but not with condensin II. This finding suggests that whatever function is played by condensin II in antagonizing sister chromatid cohesion in interphase is at least partially redundant with that played by condensin I in mitosis. Means represent three independent trials in S2R+ cells; error bars = SD; n≥53 mitotic nuclei per knockdown per trial; significance calculated for each trial using Fisher’s exact test (*, P<0.0001 in both trials; ~, P = 0.1914 in one trial and P = 0.0111 in another). Mitotic index for each knockdown, determined as the percentage of pH3-positive nuclei, is shown beneath the graph (n≥850 per genotype). (TIF) [file pgen.1006169.s013.tif]

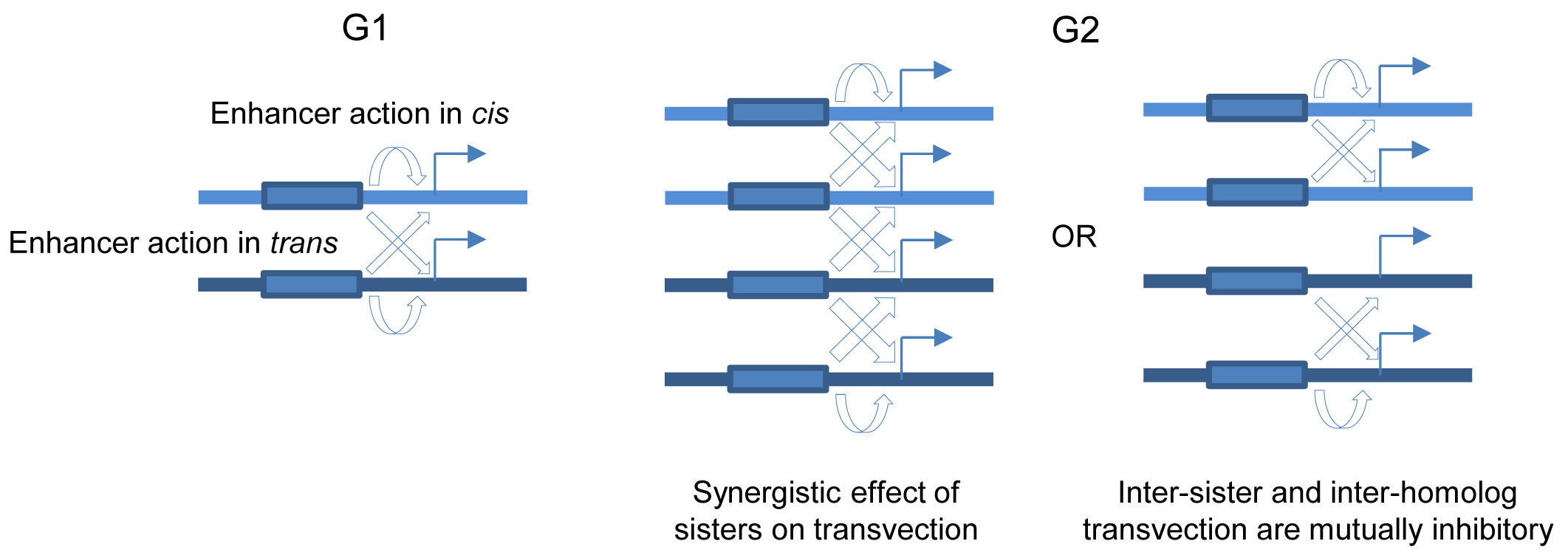

Supplement: S14 Fig — (TIF) [file pgen.1006169.s014.tif]

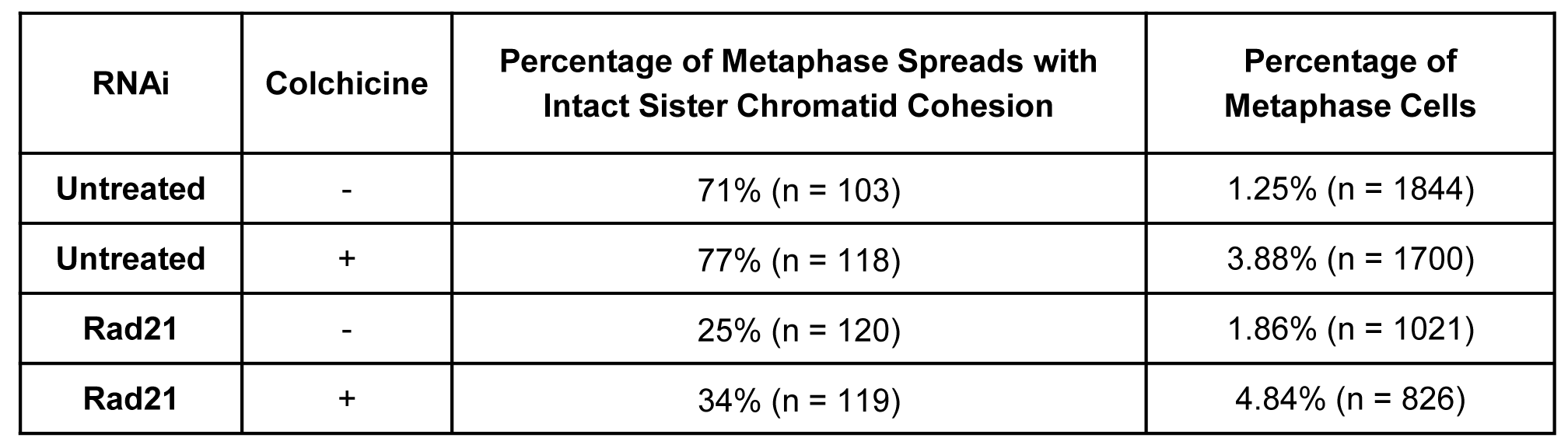

Supplement: S1 Table — Metaphase spreads were scored as either having intact cohesion (all visible chromatids were attached to a sister chromatid) or not (unattached chromatids were visible, indicative of premature loss of sister chromatid cohesion). The addition of colchicine does not significantly affect the percentage of cells with intact cohesion following Rad21 knockdown (P = 0.1570) or in cells untreated with dsRNA (P = 0.3553). Even after colchicine treatment, the percentage of cells with intact cohesion is significantly reduced following Rad21 knockdown compared to cells untreated with dsRNA (P<0.0001). (TIF) [file pgen.1006169.s015.tif]
